# Phosphorylation of eIF2α suppresses the impairment of GSH/NADPH homeostasis and mitigates the activation of cell death pathways, including ferroptosis, during ER stress

**DOI:** 10.1016/j.mocell.2025.100210

**Published:** 2025-03-13

**Authors:** Hien Thi Le, Yonghwan Kim, Mi-Jeong Kim, Seung Hwa Hyun, Hyeeun Kim, Su Wol Chung, Yeonsoo Joe, Hun Taeg Chung, Dong-Myung Shin, Sung Hoon Back

**Affiliations:** 1 School of Biological Sciences, University of Ulsan, Ulsan 44610, Korea; 2 Department of Cell and Genetic Engineering, Asan Medical Center, University of Ulsan College of Medicine, Seoul 05505, Korea; 3 College of Korean Medicine, Daegu Haany University, Gyeongsan 38610, Korea; 4 Basic-Clinical Convergence Research Center, University of Ulsan, Ulsan 44610, Korea

**Keywords:** ATF4, EIF2α phosphorylation, ER stress, Ferroptosis, Glutathione

## Abstract

eIF2α Phosphorylation helps maintain cellular homeostasis and overcome endoplasmic reticulum (ER) stress through transcriptional and translational reprogramming. This study aims to elucidate the transcriptional regulation of glutathione (GSH) and nicotinamide adenine dinucleotide phosphate hydrogen (NADPH) homeostasis through eIF2α phosphorylation and its impact on cell death during ER stress. eIF2α phosphorylation-deficient (*A/A*) cells exhibited decreased expression of multiple genes involved in GSH synthesis and NADPH production, leading to an exacerbated depletion of both cellular and mitochondrial GSH, as well as mitochondrial NADPH, during ER stress. Impaired GSH homeostasis resulted from deficient expression of ATF4 and/or its dependent factor, Nrf2, which are key transcription factors in the antioxidant response during ER stress. In contrast, the exacerbation of NADPH depletion may primarily be attributed to the dysregulated expression of mitochondrial serine-driven 1-carbon metabolism pathway genes, which are regulated by an unidentified eIF2α phosphorylation-dependent mechanism during ER stress. Moreover, the eIF2α phosphorylation-ATF4 axis was responsible for upregulation of ferroptosis-inhibiting genes and downregulation of ferroptosis-activating genes upon ER stress. Therefore, ER stress strongly induced ferroptosis of *A/A* cells, which was significantly inhibited by treatments with cell-permeable GSH and the ferroptosis inhibitor ferrostatin-1. ATF4 overexpression suppressed impairment of GSH homeostasis in *A/A* cells during ER stress by promoting expression of downstream target genes. Consequently, ATF4 overexpression mitigated ferroptosis as well as apoptosis of *A/A* cells during ER stress. Our findings underscore the importance of eIF2α phosphorylation in maintaining GSH/NADPH homeostasis and inhibiting ferroptosis through ATF4 and unidentified eIF2α phosphorylation-dependent target(s)-mediated transcriptional reprogramming during ER stress.

## Graphical abstract

**Figure:**
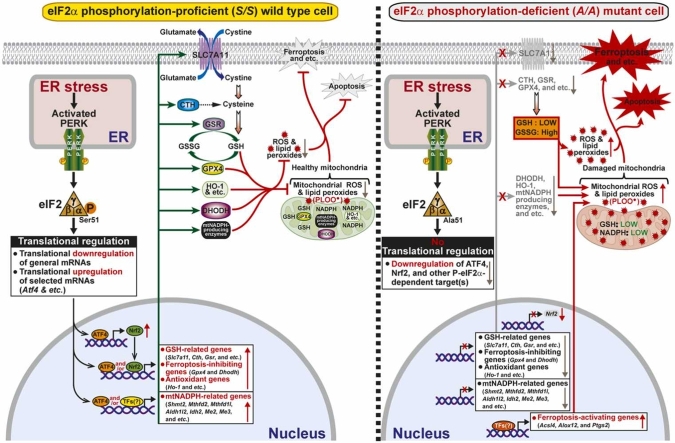

## INTRODUCTION

Multiple physiological and pathological conditions can perturb endoplasmic reticulum (ER) functions, including protein folding and glycosylation, leading to a condition referred to as “ER stress” (Marciniak and Ron, 2006). To deal with ER stress conditions, eukaryotic cells have evolved a set of signal transduction pathways called the unfolded protein response (UPR) to alter transcriptional and translational programs (Han and Kaufman, 2017). The UPR is initiated upon activation of the ER sensor inositol-requiring enzyme (IRE1), which leads to unconventional splicing of *Xbp1* mRNA encoding the X-box-binding protein 1 (XBP1) transcription factor (TF) (Han and Kaufman, 2017, Walter and Ron, 2011). Spliced XBP1 (XBP1s) induces transcription of genes encoding proteins that facilitate protein folding, secretion, and degradation in response to ER stress (Han and Kaufman, 2017, Walter and Ron, 2011). Two additional signaling branches of the UPR are mediated by the transmembrane stress sensors activating transcription factor 6 (ATF6) and protein kinase R (PKR)-like ER kinase (PERK). During ER stress, the cleaved N-terminal cytosolic domain generated by proteolytic cleavage of ATF6 at the Golgi apparatus translocates into the nucleus, where it activates target genes that encode proteins with functions in ER protein folding, ER-associated degradation, autophagy, protein secretion, and ER biogenesis (Dang et al., 2023, Han and Kaufman, 2017, Walter and Ron, 2011). Upon PERK activation, attenuation of general mRNA translation via eukaryotic translation initiation factor 2α (eIF2α) phosphorylation reduces the protein burden in the ER. Paradoxically, eIF2α phosphorylation increases the translation of specific genes, including *Atf4* (Young and Wek, 2016). ATF4 induces transcriptional expression of genes involved in redox homeostasis, amino acid metabolism, autophagy, protein folding, and protein synthesis (Walter and Ron, 2011, Young and Wek, 2016). In addition, eIF2α phosphorylation mediates maximal expression of XBP1s protein by stabilizing its mRNA (Majumder et al., 2012) and proteolytic activation of ATF6 by facilitating its trafficking from the ER to the Golgi during ER stress (Dang et al., 2023, Teske et al., 2011). Therefore, eIF2α phosphorylation, which regulates all 3 UPR pathways, helps maintain cellular homeostasis and overcome cellular stresses through transcriptional and translational reprogramming.

Under ER stress conditions, reactive oxygen species (ROS) can be generated by several cellular mechanisms, including oxidative protein folding in the ER, mitochondrial respiration, and the NOX4 nicotinamide adenine dinucleotide phosphate hydrogen (NADPH) oxidase complex (Ong and Logue, 2023, Santos et al., 2009). However, several antioxidant responses are induced in ER-stressed cells to restore redox homeostasis. During ER stress, PERK has been reported to phosphorylate and activate nuclear factor erythroid 2-related factor 2 (NFE2L2/Nrf2), a key TF for the antioxidant response (Cullinan et al., 2003, Gureev et al., 2019). PERK-mediated phosphorylation stabilizes Nrf2 protein, enabling cells to adapt to oxidative stress during ER stress (Cullinan et al., 2003). In addition, it has been proposed that ATF4 supports oxidative stress resistance by upregulating genes involved in glutathione (GSH) biosynthesis (Harding et al., 2003) and NADPH production (Wang et al., 2022, Xia et al., 2019), including amino acid transport and synthesis (glycine, cysteine, and serine) (DeNicola et al., 2015; Harding et al., 2003; Wang et al., 2022). GSH acts as a potent antioxidant to protect cells against electrophilic and oxidant species and as a cofactor for antioxidant and detoxification enzymes such as glutathione peroxidases (GPXs) and glutathione S-transferases. NADPH is an essential cofactor that regulates the reduction of cystine, the oxidized form of cysteine, and the recycling of glutathione (GSH/GSSG). It reduces oxidized glutathione (GSSG) to its reduced form (GSH) via glutathione reductase (GSR) and reduces oxidized thioredoxin through thioredoxin reductase. These processes are vital for protecting cells from oxidative stress and maintaining redox balance (Hine and Mitchell, 2012, Ribas et al., 2014). Consistently, PERK- or ATF4-deficient mouse embryonic fibroblasts (MEFs) exhibit reduced expression of glycine transporter 1 (*Glyt1*), the heavy chain of the x^−^_c_ cystine/glutamate exchanger (*Slc3a2*), light chain of x^−^_c_ cystine/glutamate exchanger (*Slc7a11*), and cystathionine gammalyase (*Cth*), all of which are important for GSH synthesis (Dickhout et al., 2012, Harding et al., 2003, Lewerenz and Maher, 2009, Zhang et al., 2022a). It is known that several metabolic pathways and enzymes contribute to NADPH production (Ju et al., 2020). Notably, NADPH is primarily generated by the pentose phosphate pathway (PPP), malic enzymes, and serine-driven 1-carbon metabolism (Fan et al., 2014). Recent studies suggest that ATF4 is an upstream TF that regulates the expression of multiple genes in the PPP, including glucose-6-phosphate dehydrogenase (*G6pdx*) and 6-phosphogluconate dehydrogenase (*6pdg*), as well as genes involved in the serine-driven 1-carbon metabolism pathway, such as phosphoglycerate dehydrogenase (*Phgdh*), phosphoserine aminotransferase 1 (*Psat1*), mitochondrial serine hydroxymethyltransferase 2 (*Shmt2*), mitochondrial methylenetetrahydrofolate dehydrogenase 2 (*Mthfd2*), and methylenetetrahydrofolate dehydrogenase 1-like (*Mthfd1l*) (Wang et al., 2022, Xia et al., 2019). However, it is not studied whether the expression of these genes is regulated by eIF2α phosphorylation during ER stress.

Several groups, including ours, reported that PERK-, eIF2α phosphorylation-, or ATF4-deficient cells exhibit increased sensitivity to oxidative stress under ER stress conditions (Back et al., 2009, Choi et al., 2017, Dickhout et al., 2012, Harding et al., 2003, Lewerenz and Maher, 2009). Ferroptosis, a newly discovered type of nonapoptotic cell death, was recently reported to be triggered by conditions under which GSH biosynthesis, the GSH-dependent antioxidant enzyme GPX4, or NADPH production is inhibited (Cao and Dixon, 2016, Du et al., 2024, Fang et al., 2023, Jiang et al., 2021, Mo et al., 2024b, Stockwell, 2022, Zhang et al., 2023). Ferroptosis is characterized by the accumulation of lethal lipid peroxides under iron- and ROS-rich conditions. Therefore, eIF2α phosphorylation may play an important role to prevent ferroptosis in response to ER stress. However, the molecular mechanisms involved in prevention and regulation of ferroptosis through eIF2α phosphorylation remain unclear.

In the present study, we found that ATF4 overexpression (OE) in *A/A* cells prevents ROS accumulation and significantly reduces the exacerbation of GSH depletion under ER stress in both cellular and mitochondrial contexts by increasing the expression of multiple antioxidant genes, including those involved in GSH synthesis. Conversely, eIF2α wild-type (WT) OE, but not ATF4 OE, mitigates the exacerbation of NADPH depletion by inhibiting the dysregulated expression of several mitochondrial NADPH-producing genes in *A/A* cells during ER stress. Consequently, ATF4 OE mitigated ferroptosis as well as apoptosis induced by eIF2α phosphorylation deficiency during ER stress. Our data demonstrate that eIF2α phosphorylation and its downstream targets, ATF4 and unidentified gene(s), maintain redox homeostasis (especially GSH and NADPH homeostasis) and inhibit ferroptosis by mediating transcriptional reprogramming under ER stress conditions.

## MATERIALS AND METHODS

### Cell Culture

All cell lines were incubated at 37°C with 5% CO_2_ in a humidified incubator. Wild-type (*S/S*) and eIF2α phosphorylation-deficient (*A/A*) MEFs (Dang et al., 2023) were cultured in Dulbecco’s modified Eagle’s medium (DMEM; Thermo Fisher Scientific, 11995065) supplemented with 10% FBS (WelGENE, S001-01), 1% penicillin-streptomycin (WelGENE, LS202-02), and 1% MEM nonessential amino acids (NEAAs; Thermo Fisher Scientific, 11140-050). Immortalized hepatocytes (*S/S*^*Hep*^ and *A/A*^*Hep*^) (Dang et al., 2023) were cultured in Medium 199 (WelGENE, LM006-01) supplemented with 10% FBS and 1% penicillin-streptomycin. Wild-type (*Atf4*^*+/+*^) and ATF4-knockout (*Atf4*^*-/-*^) MEFs were cultured in DMEM supplemented with 10% FBS, 1% penicillin-streptomycin, 2% MEM amino acids (WelGENE, LS004-01), 1% MEM NEAAs (WelGENE, LS005-01), and 55 µM β-mercaptoethanol (Sigma-Aldrich, M3148), as previously described (Han et al., 2013).

### Expression Vectors

The empty control, ATF4-/EGFP-expressing, and wild-type eIF2α-expressing adenoviral vectors (*pShuttle-CMV*, *pAD-Track-ATF4*, and *pShuttle-CMV-eIF2α[WT]*) were described previously (Dang et al., 2023). The empty control and ATF4-expressing vectors (*pCGN-Vec* and *pCGN-ATF4-IRES-EGFP*) were described previously (Dang et al., 2023). *pGL4.37-ARE-firefly luciferase* (Promega, E3641) contains 4 copies of an antioxidant response element (ARE) that drives transcription of the luciferase reporter gene *luc2P* (*Photinus pyralis*). The *pRL-CMV* plasmid expressing CMV promoter-driven *Renilla* luciferase was purchased from Promega (E2261).

To generate a plasmid expressing both hemagglutinin (HA)-tagged ATF4 and the nuclear-localized near-infrared fluorescent protein iRFP713 (HA-ATF4/iRFP713-Nuc) (*pCMV-HA-ATF4-IRES-iRFP713-Nuc*), the *pCMV-iRFP713-Nuc* empty vector, which expresses iRFP713 using an internal ribosome entry site (IRES), was first constructed by replacing the *AcGFP* gene of *pAcGFP-Nuc* (Clontech, 632431) with the *iRFP713* gene of *piRFP* (Addgene, 31857). The cDNA fragment encoding iRFP713 was amplified from *piRFP* via PCR. The PCR product treated with *Age*I and *Bgl*II was inserted into *pAcGFP-Nuc* treated with the same restriction enzymes to construct *pCMV-iRFP713-Nuc*. Next, the IRES-driven iRFP713-Nuc-expressing plasmid (*pCMV-IRES-iRFP713-Nuc*) was constructed by inserting the IRES sequence into *pCMV-iRFP713-Nuc*. To construct *pCMV-IRES-iRFP713-Nuc*, the IRES DNA fragment was amplified from *pCGN-IRES-EGFP* (Dang et al., 2023) via PCR. The PCR product treated with *Nhe*I and *Age*I was inserted into *pCMV-iRFP713-Nuc* treated with the same restriction enzymes. To construct *pCMV-HA-ATF4-IRES-iRFP713-Nuc*, the *Nhe*I-Klenow-*Nde*I fragment containing CMV-HA-ATF4 from *pCGN-ATF4* (Dang et al., 2023) was inserted into *pCMV-IRES-iRFP713-Nuc* treated with AfeI and *Nde*I.

To generate a plasmid expressing both 3xFLAG-tagged eIF2α(WT) and iRFP713-Nuc (FLAG-eIF2α/iRFP713-Nuc) (*pCMV-3xFlag-eIF2α-IRES-iRFP713-Nuc*), the *pCMV-3xFlag-eIF2α(WT)* plasmid was first constructed. To construct *pCMV-3xFlag-eIF2α(WT)*, the cDNA fragment encoding WT eIF2α was transferred into *p3xFlag-CMV-10* (Sigma-Aldrich, 32190102) to add an N-terminal 3xFLAG tag. The cDNA fragment encoding eIF2α(WT) was amplified from *pShuttle-CMV-eIF2α(WT)* (Dang et al., 2023) via PCR. The PCR product treated with *Hin*dIII and *Eco*RI was inserted into *p3xFlag-CMV-10* treated with the same restriction enzymes to construct *pCMV-3xFlag-eIF2α(WT)*. To construct *pCMV-3xFlag-eIF2α-IRES-iRFP713-Nuc*, the fragment containing *CMV-3xFlag-eIF2α(WT)* generated by digesting *pCMV-3xFlag-eIF2α(WT)* with *Nde*I and *Sma*I was inserted into *pCMV-IRES-iRFP713-Nuc* treated with *Nhe*I-Klenow-*Nde*I. The PCR primer pairs used in this study are listed in Table S1.

### Recombinant Adenovirus Production

Recombinant adenoviruses expressing ATF4/EGFP or eIF2α(WT) using the AdEasy vector system (Agilent Technologies, 240009) were generated using a previously described procedure (Dang et al., 2023). The indicated MEFs were seeded into 100-mm culture dishes at a density of 7 × 10^5^ cells/dish or into 96-well culture dishes at a density of 6,000 cells/well, cultured for 12 to 16 h, and infected with the indicated recombinant adenoviruses (*Ad-vector*, *Ad-ATF4/EGFP*, or *Ad-eIF2α[WT]*) for 24 h. The infected cells were treated with or without tunicamycin (Tm) for the indicated durations before undergoing various analyses, including quantitative PCR (qPCR), western blotting, mitochondrial function assessments, oxidative stress marker assessments, and cell viability assays.

### Quantitative PCR

After the indicated treatments, total RNA was isolated from cells using QIAzol Lysis Reagent (QIAGEN, QI-79306). cDNA was synthesized using a High-Capacity cDNA RT Kit (Applied Biosystems, ABS-4368814). qPCR was performed using TOPreal SYBR Green qPCR High-ROX PreMIX (Enzynomics, RT501M) and a StepOnePlus Real-Time PCR System (Applied Biosystems). The specificity of each primer pair was confirmed by melting curve analysis. The levels of target mRNAs were normalized to that of *β-actin* (*β-act*) mRNA. The qPCR primer pairs used in this study are listed in Table S2.

### Western Blot (WB) Analysis

Cells were lysed in Nonidet P40 lysis buffer (1% IGEPAL CA-630 NP40, 50 mM Tris-HCl, pH 7.5, 150 mM NaCl, 0.05% sodium dodecyl sulfate [SDS], 0.5 mM sodium orthovanadate, 100 mM NaF, 50 mM β-glycerophosphate, and Halt Protease Inhibitor Cocktail). Cell lysates were centrifuged at 13,000 ×g for 15 min at 4°C, and the supernatants were collected. For WB analysis of NFE2L2/Nrf2, cells were treated with Tm for the indicated durations and then with MG132 (20 µM) for 1 h before harvesting samples. Cells were directly lysed in SDS lysis buffer (1% SDS, 50 mM Tris-Cl, pH 7.5, 150 mM NaCl, 0.5 mM sodium orthovanadate, 100 mM NaF, and 50 mM β-glycerophosphate) supplemented with Halt Protease Inhibitor Cocktail (Thermo Fisher Scientific, 1861279). The lysates were immediately heated for 15 min at 100°C. The homogenates were centrifuged at 13,000g for 15 min at 4°C, and the supernatants were collected. Protein concentrations were determined using a Pierce BCA Protein Assay Kit (Thermo Fisher Scientific, 23227). Cell lysates were subjected to WB analysis, as described previously (Dang et al., 2023). Information regarding antibodies is provided in Table S3.

### Dual-Luciferase Assay

The luciferase assay was performed to assess the transcriptional activity of NFE2L2/Nrf2. *S/S* and *A/A* MEFs were seeded into 6-well plates at a density of 2.5 × 10^5^ cells/well and cultured overnight. Both *pGL4.37-ARE-firefly luciferase* (for 4xARE motif-driven firefly luciferase) and *pRL-CMV* (for CMV promoter-driven *Renilla* luciferase) plasmids were transfected using Mirus Bio TransIT-LT1 transfection reagent (Fisher Scientific, MIR2306). CMV promoter-driven *Renilla* luciferase was used to normalize the transfection and expression efficiencies. If necessary, the other indicated constructs (*pCGN-Vec* or *pCGN-ATF4-IRES-EGFP*) were also co-transfected for 24 h according to the manufacturer’s instructions. After treatment with Tm (1 µg/ml) for the indicated durations, cells were washed once with phosphate-buffered saline (PBS) and harvested for luciferase assays using the Dual-Luciferase Assay System (Promega, E1980) according to the manufacturer’s instructions. Chemiluminescent signals were measured using a SpectraMax ID3 microplate reader (Molecular Devices). Firefly luciferase activity was normalized by *Renilla* luciferase activity in each sample. The presented data were replicated in at least 3 independent experiments.

### Cell Imaging Using Confocal Microscopy

Cells were plated on collagen-coated 35-mm glass-bottom confocal dishes (SPL Life Science, 101350) at a density of 2.5 × 10^5^ cells/dish. The next day, cells were treated with vehicle (Veh, DMSO) and either Tm (1 µg/ml) or thapsigargin (Tg, 500 nM) in phenol-red free DMEM (Gibco, 21063029) for the indicated durations. In ATF4 or eIF2α(WT) OE experiments, cells were infected with the indicated recombinant adenoviruses (*Ad-vector*, *Ad-ATF4/EGFP*, or *Ad-eIF2α[WT]*) for 24 h before Tm treatment. During the last 30 min of the chemical treatment, the cell culture medium was supplemented with MitoSOX Red (5 µM, Invitrogen, M36008), CellROX Deep Red (5 µM, Invitrogen, C10422), BODIPY 581/591 C11 (2.5 µM, Invitrogen, D3861), or MitoPeDPP (0.2 µM, Dojindo, M466) to stain cells. If nuclear staining was necessary, Hoechst 33258 (5 µg/ml, Sigma-Aldrich, 94403) was added together with the dye. Live-cell imaging was performed using a FV1200-OSR confocal microscope (Olympus). The staining intensity was measured using the mean fluorescence intensity tool of FV10-ASW-4.2 software (Olympus).

### GSH/Glutathione Disulfide (GSSG) Assay

Cells were plated in 150-mm culture dishes at a density of 2 × 10^6^ cells/dish, cultured for longer than 12 h, and treated with Veh (DMSO) and either Tm (1 µg/ml) or Tg (500 nM) for 24 h. In ATF4 or eIF2α(WT) OE experiments, cells were infected with the indicated recombinant adenoviruses (*Ad-vector*, *Ad-ATF4/EGFP*, or *Ad-eIF2α[WT]*) for 24 h before Tm treatment. For the mitochondrial GSH/GSSG assay, mitochondrial fractions were isolated using a Mitochondrial Isolation Kit (Abcam, 89874) according to the manufacturer’s instructions. Upon harvesting cells or mitochondrial fractions, the levels of GSH and GSSG were measured using a GSH/GSSG Quantification Kit (Dojindo Molecular Technologies, T41910) according to the manufacturer’s instructions. The GSH and GSSG concentrations were calculated using a standard curve and normalized to the total protein level.

### Imaging and Monitoring of the Mitochondrial GSH Level

Real-time assessment of mitochondrial GSH levels in individual live cells was performed, as described previously (Jeong et al., 2018, Kim et al., 2023). The assay enabled nondestructive, comprehensive, and image-based high-throughput evaluations of both qualitative and quantitative aspects of GSH dynamics in mitochondria of live cells. It leveraged the distinct characteristics of fluorescent real-time thiol tracer (FreSHtracer), a reversible chemical probe specifically designed for cellular GSH. Upon interaction with cellular GSH, FreSHtracer exhibited a spectral shift in the λmax of its ultraviolet-visible absorption, transitioning from 520 to 430 nm. This shift resulted in a decrease in yellow fluorescence intensity at 580 nm (F580, λex 520 nm) and a corresponding increase in green fluorescence intensity at 510 nm (F510, λex 430 nm). Consequently, to determine the fluorescence ratios of FreSHtracer, fluorescence emissions were quantified at 510 and 580 nm following excitation at 430 and 520 nm, respectively.

To monitor GSH levels in mitochondria, cells were labeled with MitoFreSHtracer (10 µM), a derivative of FreSHtracer that targets mitochondria due to attachment of a triphenylphosphonium moiety (Jeong et al., 2018), for the last 1 h of Tm treatment. Fluorescence signals of MitoFreSHtracer were continuously recorded in live cells using FreSHcell Q (Cell2in), an automated high-content live-cell imaging system equipped with an sCMOS camera. Imaging was performed at 100× magnification in accordance with the manufacturer's guidelines. The acquired fluorescence images were analyzed using NIS-Elements AR software from Nikon (Minato-ku). Each fluorescence image underwent processing, commencing with reconstitution for cell segmentation via the rolling ball method, to correct the background intensity. This was followed by shading correction to rectify illumination in homogeneities within the images. The artificial intelligence module integrated into NIS-Elements AR software was then employed to distinguish cells from the background and to facilitate segmentation.

### Electroporation of Plasmids

To express iRFP713-Nuc, FLAG-eIF2α/iRFP713-Nuc, or HA-ATF4/iRFP713-Nuc in *A/A* MEFs via electroporation, cells grown to 80% confluency were harvested. After 2 washes, *A/A* MEFs were resuspended in Resuspension Buffer R from a Neon transfection kit (Thermo Fisher Scientific, MPK1025) at a density of 5.0 × 10^6^ cells/ml. The resuspended cells were mixed with a 10-µl Neon tip (Thermo Fisher Scientific, MPK1025) and 20 µg of the Vec-, iRFP713-Nuc-, FLAG-eIF2α/iRFP713-Nuc-, or HA-ATF4/iRFP713-Nuc-expressing plasmid. These mixtures were then exposed to a high-voltage electrical pulse (1350 V, 30 ms, 1 pulse) using a Neon electroporation device (Thermo Fisher Scientific). Electroporated cells were grown in complete media for 48 h and treated with or without Tm for 24 h before undergoing mitochondrial GSH analysis with MitoFreSHtracer, iRFP713 expression analysis with a flow cytometer, or WB analysis.

### Measurement of Total NADP(H) Amounts

Cells were plated the day before in 96-well plates at a density of 8,000 cells/well. The next day, cells were treated with Veh (DMSO) or Tm (1 µg/ml) for the indicated durations. In ATF4 or eIF2α(WT) OE experiments, *A/A* MEFs were infected with the indicated recombinant adenoviruses (*Ad-vector*, *Ad-ATF4/EGFP*, or *Ad-eIF2α[WT]*) for 24 h before Tm treatment. Total NADP(H) amounts were measured using NADPH/NADP^+^ Glo-Assay (Promega, G9081) following the manufacturer's instructions. Chemiluminescent signals were measured using a SpectraMax ID3 microplate reader (Molecular Devices). The levels were normalized to the protein concentrations. The presented data were replicated in at least 3 independent experiments.

### Flow Cytometric Analysis of Annexin V- and 7-Aminoactinomycin D (7-AAD)-Stained Cells

Apoptotic cell death and other forms of cell death involving the loss of plasma membrane integrity were detected by staining cells with annexin V-PE and 7-AAD (Thermo Fisher Scientific, 88-8102-72) according to the manufacturer’s instructions. Cells were plated in 6-well plates at a density of 2.5 × 10^5^ cells/well and cultured for longer than 12 h. In the case of Z-VAD, GSH-EE, and ferrostatin-1 (Fer-1) treatments, cells were pretreated with Veh (-), Z-VAD (20 µM), GSH-EE (0.75 µM), Fer-1 (5 µM), Z-VAD plus GSH-EE, or Z-VAD plus Fer-1 for 1 h. Cells were then treated with Tm (1 µg/ml) or Tm plus other chemicals for the indicated durations. In ATF4 or eIF2α(WT) OE experiments, cells were infected with the indicated recombinant adenoviruses (*Ad-vector*, *Ad-ATF4/EGFP*, or *Ad-eIF2α[WT]*) for 24 h before Tm treatment. Finally, cells were harvested and analyzed using a FACSCanto II flow cytometer (BD Biosciences) and FlowJo software (Ashland). The results are presented as dot plots.

### Cell Counting Kit-8 (CCK-8) Assay

The CCK-8 assay (Dojindo, CK04) was performed according to the manufacturer’s instructions. Cells were seeded into 96-well plates at a density of 8,000 cells/well, cultured for longer than 12 h, and treated with the specified chemicals for the indicated durations. After treatment, CCK-8 reagent (10 µl) was added to each well. After incubation for 2 h in a CO_2_ incubator, the conversion of CCK-8 reagent to chromogenic formazan was monitored with a SpectraMax ID3 microplate reader at a wavelength of 450 nm.

### Lactate Dehydrogenase (LDH) Cytotoxicity Assay

Cytotoxicity was evaluated by measuring the amount of LDH that leaked into the culture medium using an LDH Cytotoxicity Colorimetric Assay Kit (BioVision, K311-400). Cells were seeded into a 96-well plate at a density of 8,000 cells/well, cultured for longer than 12 h, and treated with the specified chemicals for the indicated durations. After treatment, cell supernatants were collected and released LDH was analyzed using the assay kit according to the manufacturer’s instruction. Absorbance at a wavelength of 450 nm was measured using a SpectraMax ID3 microplate reader.

### Flow Cytometric Analyses of Cells Stained With Propidium Iodide (PI), BODIPY 581/591 C11, and MitoPeDPP

Cells were seeded into 6-well plates at a density of 2.5 × 10^5^ cells/well, cultured for longer than 12 h, treated with the specified chemicals for the indicated durations, harvested, and washed twice with cold PBS. For ATF4 OE experiments, *A/A* MEFs were transfected for 24 h with either the *pCMV-IRES-iRFP713-Nuc* or *pCMV-HA-ATF4-IRES-iRFP713-Nuc* plasmid using TransIT-LT1 transfection reagent (MIR2305, Mirus). They were then treated with or without Tm for an additional 24 h. Treated cells were harvested and washed twice with cold PBS. Next, cells were stained with PI (2 µg/ml, Sigma-Aldrich, P4864) for 15 min, a lipid peroxidation probe (BODIPY 581/591 C11, 2.5 µM, Invitrogen, D3861) for 30 min, or a mitochondrial lipid peroxidation probe (MitoPeDPP, 0.2 µM, Dojindo, M466) for 30 min in the dark. Finally, cells were washed 3 times with PBS to remove unincorporated dyes and resuspended in 300 µl of PBS. Cells were then analyzed using a FACSCanto II flow cytometer (BD Biosciences) and FlowJo software (Ashland). The results are presented as dot plots, bar graphs, or histograms.

### Malondialdehyde (MDA) Assay

The relative concentrations of MDA (an end product of lipid peroxidation) in cell lysates were measured using a Lipid Peroxidation (MDA) Assay Kit (Abcam, ab118970) following the manufacturer’s instructions. Cells were plated in 150-mm culture dishes at a density of 2 × 10^6^ cells/dish, cultured for longer than 12 h, and treated with the specified chemicals for the indicated durations. Briefly, thiobarbituric acid was reacted with MDA in the samples at 95°C, and the MDA-TBA adduct was fluorometrically measured (excitation wavelength of 530 nm and emission wavelength of 550 nm) using a SpectraMax ID3 microplate reader. The MDA levels were normalized to the protein concentrations.

### Statistical Analysis

All data are presented as mean ± standard error of the mean (SEM). All experiments were performed at least 3 times. Statistical analyses were performed using Student’s t-test, a 1-way analysis of variance (ANOVA), or a 2-way ANOVA with GraphPad Prism 8.4.3 (GraphPad Software). Statistical significance is indicated in the figures (*^,#,&^*P* < .05, **^,##,&&^*P* < .01, ***^,###,&&&^*P* < .001).

## RESULTS

### eIF2α Phosphorylation Prevents Dysregulation of Cytosolic and Mitochondrial Redox Homeostasis (Especially GSH Homeostasis) by Maintaining Optimal Expression of Antioxidant Genes Including GSH-Related Genes During ER Stress

**Fig. 1:**
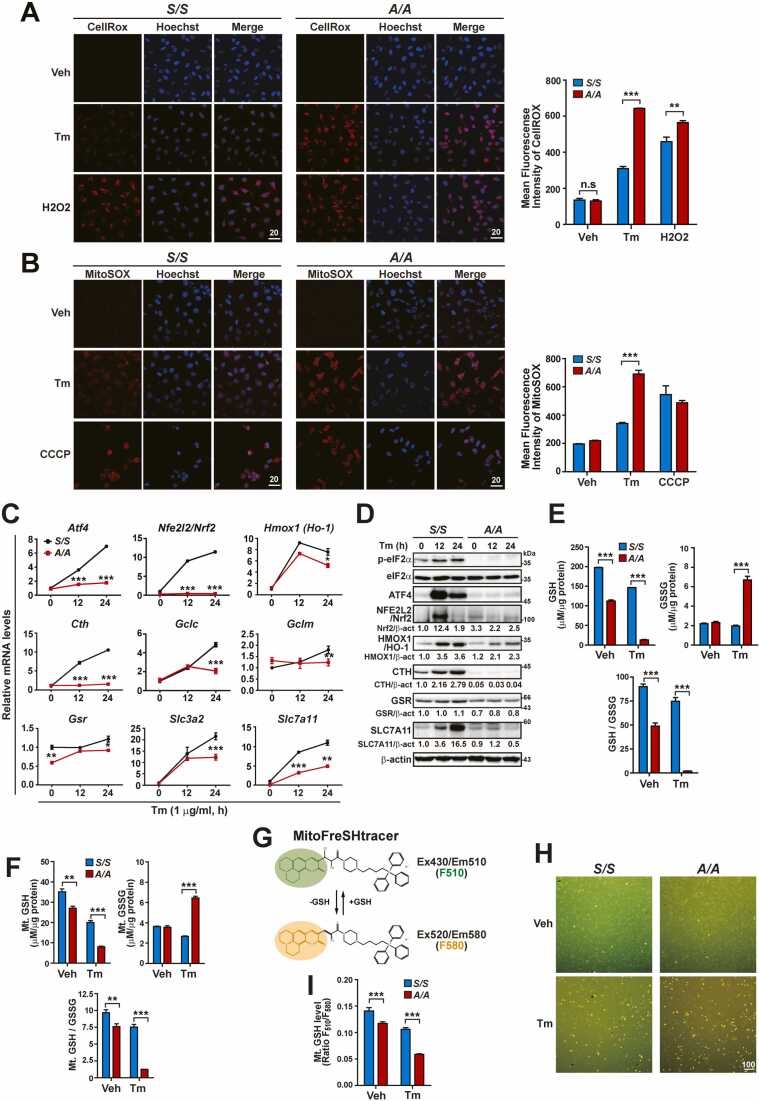
eIF2α phosphorylation deficiency dysregulates expression of ROS defense genes, including GSH-related genes, decreases cellular and mitochondrial GSH levels, and thereby induces accumulation of cellular and mitochondrial ROS during ER stress. (A) Representative CellROX staining images of *S/S* and *A/A* MEFs. Cells were treated with Veh (DMSO, 24 h), Tm (1 µg/ml, 24 h), or H_2_O_2_ (1.5 mM, 12 h) and stained with CellROX Deep Red (red) and Hoechst 33258 (blue) for the last 30 min. H_2_O_2_ was used as a cellular ROS-inducing agent. Scale bar: 20 µm. The graph depicts quantification of the mean fluorescence intensity (MFI) of CellROX. Data are presented as mean ± SEM (n = 3, 15 random fields per condition). **P < .01 and ***P < .001, *S/S* versus *A/A*. (B) Representative MitoSOX staining images of *S/S* and *A/A* MEFs. Cells were treated with Veh (DMSO, 24 h), Tm (1 µg/ml, 24 h), or carbonyl cyanide-m-chlorophenyl hydrazone (CCCP, 4 µM, 4 h) and stained with MitoSOX Red (red) and Hoechst 33258 (blue) for the last 30 min. CCCP was used as a mitochondrial ROS-inducing agent. Scale bar: 20 µm. The graph depicts quantification of the MFI of MitoSOX. Data are presented as mean ± SEM (n = 3, 15 random fields per condition). ***P < .001, *S/S* versus *A/A*. (C) Quantitative RT-PCR analysis of mRNA levels of ROS defense genes, including GSH-related genes in *S/S* and *A/A* MEFs treated with Tm for the indicated durations. Data are presented as mean ± SEM (n = 3). *P < .05, **P < .01, and ***P < .001, *S/S* versus *A/A* at each time point. (D) WB analysis of ROS defense proteins in lysates of *S/S* and *A/A* MEFs treated with Tm for the indicated durations. Protein levels normalized by β-act levels are shown below the panels. (E) Quantification of cellular GSH and GSSG levels in *S/S* and *A/A* MEFs treated with Veh or Tm for 24 h. Data are presented as mean ± SEM (n = 3). ***P < .001, *S/S* versus *A/A*. (F) Quantification of mitochondrial (Mt) GSH and GSSG levels in mitochondria-enriched fractions of *S/S* and *A/A* MEFs treated with Veh or Tm for 24 h. Data are presented as mean ± SEM (n = 3). **P < .01 and ***P < .001, *S/S* versus *A/A*. (G) Structure of MitoFreSHtracer and changes in its fluorescence spectra upon reaction with GSH. (H) Representative merged images of F_510_ (green) and F_580_ (yellow) fluorescence in MitoFreSHtracer-loaded living *S/S* and *A/A* MEFs. Cells were treated with Veh or Tm for 24 h and stained with MitoFreSHtracer for the last 1 h. Scale bar: 100 µm. (I) A graph depicting quantification of mitochondrial GSH levels (MitoFreSHtracer fluorescence ratio F_510_/F_580_) in *S/S* and *A/A* MEFs treated with Veh or Tm for 24 h. Data are presented as mean ± SEM (n = 6). ***P < .001, *S/S* versus *A/A*.

It is believed that ER stress generates ROS via cellular processes, including oxidative protein folding and mitochondrial respiration (Ong and Logue, 2023, Santos et al., 2009). Therefore, we assessed how eIF2α phosphorylation helps cells to cope with ER stress-induced ROS. Microscopic observations of intracellular ROS accumulation using CellROX Deep Red and mitochondrial ROS accumulation using MitoSOX Red revealed a markedly increased level of both intracellular and mitochondrial ROS in *A/A* cells (homozygous S51A mutation at the phosphorylation site in eIF2α) compared with *S/S* cells (wild type) under Tm- and Tg-treated conditions, although *S/S* cells also exhibited mild ROS accumulation under ER stress conditions (Figs. 1A, 1B, S1A, S1B, S2A, and S2B). However, compared with Tm and Tg treatments, there is only a slight difference in H_2_O_2_-mediated intracellular ROS accumulation between *S/S* and *A/A* cells (Fig. 1A), while there is no significant difference in CCCP-mediated mitochondrial ROS accumulation between these two cell types (Fig. 1B). These results suggest that eIF2α phosphorylation deficiency promotes ROS accumulation during ER stress. Phosphorylation of eIF2α is required for optimal expression of many UPR genes, including *Atf4* under ER stress conditions (Fig. S3A and B) (Back et al., 2009, Dang et al., 2023, Majumder et al., 2012, Teske et al., 2011, Wek et al., 2006, Young and Wek, 2016). Among eIF2α phosphorylation-dependent genes, ATF4 is an important TF for the maintenance of redox homeostasis during ER stress (Almeida et al., 2022, Kasai et al., 2019, Quiros et al., 2017). In addition, recent reports suggest that ATF4 is responsible for the increased expression of *Nfe2l2/Nrf2* mRNA and its protein during ER stress (Kress et al., 2023, Sarcinelli et al., 2020). Consistently, NFE2L2/Nrf2 expression was impaired in *Atf4*^*-/-*^ MEFs but was strongly induced in *Atf4*^*+/+*^ MEFs under various stress conditions, such as following treatment with ER stress inducers (Tm, Tg, and DTT), H_2_O_2_ exposure, or removal of NEAAs (Fig. S4A-E). Therefore, mRNA and protein expression of not only *Atf4* but also *Nfe2l2/Nrf2* were decreased in *A/A* cells but were strongly increased in *S/S* cells under ER stress conditions (Figs. 1C, 1D, S2C, S2D, S3A, and S3B). Furthermore, activity assays of the NFE2L2/Nrf2 response element (ARE)-containing luciferase reporter showed that Nrf2-dependent reporter activities were significantly diminished in *A/A* MEFs but strongly increased in *S/S* MEFs upon Tm treatment (Fig. S4F). However, transient OE of ATF4 during ER stress restored these reporter activities in *A/A* MEFs (Fig. S4G). Thus, expression of *Nfe2l2*/*Nrf2* mRNA and its protein may be dysregulated due to impairment of ATF4 expression in eIF2α phosphorylation-deficient cells. Next, to elucidate the underlying molecular mechanism of ROS accumulation in *A/A* cells during ER stress, we assessed expression changes of ATF4- and/or NFE2L2-/Nrf2-dependent antioxidant genes (such as *Hmox1*, *Cth*, *Gclc*, *Gclm*, *Gsr*, *Slc3a2*, and *Slc7a11*) (Dickhout et al., 2012, Done and Traustadottir, 2016, Harding et al., 2003, Hayes and Dinkova-Kostova, 2014, Kasai et al., 2020, Lewerenz and Maher, 2009, Torrence et al., 2021, Ye et al., 2014). Among them, we focused on changes in the expression of GSH-related genes (*Cth*, *Gclc*, *Gclm*, *Gsr*, *Slc3a2*, and *Slc7a11*) because ATF4 and NFE2L2/Nrf2 cooperatively function to express these genes (Dickhout et al., 2012, Done and Traustadottir, 2016, Harding et al., 2003, Hayes and Dinkova-Kostova, 2014, Kasai et al., 2020, Lewerenz and Maher, 2009, Torrence et al., 2021, Ye et al., 2014). The mRNA levels of all examined genes (*Hmox1*, *Cth*, *Gclc*, *Gclm*, *Gsr*, *Slc3a2*, and *Slc7a11*) were significantly lower in *A/A* MEFs than in *S/S* MEFs at a late time point (24 h) or several time points (Fig. 1C). Similar to MEFs, mRNA levels of GSH-synthesizing genes were significantly lower in *A/A*^*Hep*^ cells than in *S/S*^*Hep*^ cells, regardless of ER stress (*Cth*, *Gclc*, *Slc3a2*, and *Slc7a11*) or specifically during ER stress (*Gclm*) (Fig. S2C). In addition, eIF2α phosphorylation deficiency decreased the protein levels (HO-1, CTH, GSR, and SLC7A11) of the examined genes (Fig. 1D and S2D). Among the examined genes, eIF2α phosphorylation deficiency most significantly affected the mRNA and protein levels of *Cth* and *Slc7a11* during ER stress (Figs. 1C, 1D, S2C, and S2D). Both genes are responsible for supplying cellular cysteine via hydrolysis of cystathionine in the trans-sulfuration pathway or import of its oxidized dipeptide, cystine (Zhang et al., 2022a). In addition, cellular cysteine is the rate-limiting precursor for the production of GSH, a prominent antioxidant in mammalian cells (Poltorack and Dixon, 2022, Zhang et al., 2022a). Therefore, we assessed the levels of GSH and GSSG in total cellular and mitochondria-enriched fractions of *S/S* and *A/A* MEFs treated with or without Tm and Tg (Figs. 1E, 1F, S1C, and S1D). Tm and Tg treatments decreased GSH levels more in *A/A* MEFs than in *S/S* MEFs, although it decreased cellular and mitochondrial GSH levels in both *S/S* and *A/A* MEFs (Figs. 1E, 1F, S1C, and S1D). In addition, measurement of mitochondrial GSH levels in living cells using the ratiometric and mitochondria-specific GSH probe MitoFreSHtracer (Jeong et al., 2018, Kim et al., 2023) confirmed that phosphorylation of eIF2α was important to maintain mitochondrial GSH levels during ER stress, although it was also necessary under normal conditions (Fig. 1G-I). Tm and Tg treatments significantly increased cellular and mitochondrial GSSG levels in *A/A* MEFs but not in *S/S* MEFs (Figs. 1E, 1F, S1C, and S1D). Consequently, cellular and mitochondrial GSH/GSSG ratios drastically decreased in *A/A* MEFs compared with *S/S* MEFs upon Tm and Tg treatments, although they were lower in *A/A* MEFs than in *S/S* MEFs before Tm treatment (Figs. 1E, 1F, S1C, and S1D). Our results suggest that eIF2α phosphorylation is required for proper expression of antioxidant genes, including GSH-synthesizing genes, and thereby prevents dysregulation of cytosolic and mitochondrial redox homeostasis (especially GSH homeostasis) during ER stress.

### ATF4 OE Increases Expression of Antioxidant Genes Including GSH-Synthesizing Genes and Thereby Ameliorates Impairment of Cellular and Mitochondrial Redox Homeostasis (Especially GSH Homeostasis) in *A/A* Cells During ER Stress

**Fig. 2:**
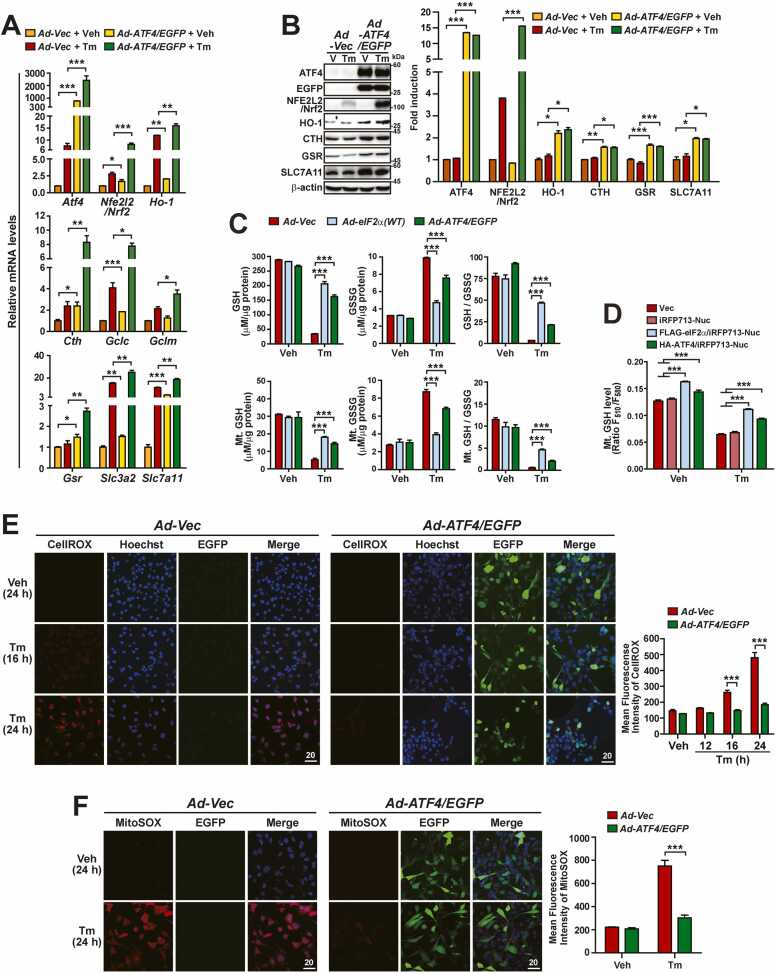
ATF4 OE promotes expression of ROS defense genes, including GSH-related genes and thereby suppresses the decreases of cellular and mitochondrial GSH levels and accumulation of cellular and mitochondrial ROS in *A/A* cells during ER stress. (A) Quantitative RT-PCR analysis of mRNA levels of ROS defense genes, including GSH-related genes in Vec- or ATF4-/EGFP-overexpressing *A/A* MEFs treated with Veh or Tm for 24 h. Data are presented as mean ± SEM (n = 3). *P < .05, **P < .01, and ***P < .001, *Vec* versus *ATF4/EGFP*. (B) WB analysis of ROS defense proteins in lysates of Vec- or ATF4-/EGFP-overexpressing *A/A* MEFs treated with Veh (V) or Tm for 24 h. The graph depicts the protein level normalized by the β-act level. Data are presented as mean ± SEM (n = 3). *P < .05, **P < .01, and ***P < .001, *Vec* versus *ATF4/EGFP*. (C) Quantification of cellular (upper panels) and mitochondrial (lower panels) GSH and GSSG levels in Vec-, eIF2α(WT)-, or ATF4-/EGFP-overexpressing *A/A* MEFs treated with Veh or Tm for 24 h. Data are presented as mean ± SEM (n = 3). ***P < .001, *Vec* versus *eIF2α(WT)* or *ATF4/EGFP*. (D) A graph depicting quantification of mitochondrial GSH levels (MitoFreSHtracer fluorescence ratio F_510_/F_580_) in Vec-, iRFP713-Nuc-, FLAG-eIF2α/iRFP713-Nuc-, or HA-ATF4/iRFP713-Nuc-overexpressing *A/A* MEFs treated with Veh or Tm for 24 h. Data are presented as mean ± SEM (n = 6). ***P < .001, *Vec* and *iRFP713-Nuc* versus *FLAG-eIF2α/iRFP713-Nuc* or *HA-ATF4/iRFP713-Nuc*. Representative merged images of MitoFreSHtracer fluorescence, the plasmid transfection efficiency, and WB analysis of overexpressed proteins for (D) are presented in Figure S5A-C, respectively. (E) Representative CellROX staining images of Vec- or ATF4-/EGFP-overexpressing *A/A* MEFs. *A/A* MEFs infected with Vec- or ATF4-/EGFP-expressing adenoviruses for 24 h were treated with Veh or Tm for the indicated durations and stained with CellROX Deep Red (red) and Hoechst 33258 (blue) for the last 30 min. Expression of ATF4 is indicated by the green fluorescence of EGFP. Scale bar: 20 µm. The graph depicts the quantification of the MFI of CellROX. Data are presented as mean ± SEM (n = 3, 15 random fields per condition). ***P < .001, *Vec* versus *ATF4/EGFP*. (F) Representative MitoSOX staining images of Vec- or ATF4-/EGFP-overexpressing *A/A* MEFs. *A/A* MEFs infected with Vec- or ATF4-/EGFP-expressing adenoviruses for 24 h were treated with Veh or Tm for 24 h and stained with MitoSOX Red (red) and Hoechst 33258 (blue) for the last 30 min. Expression of ATF4 is indicated by the green fluorescence of EGFP. Scale bar: 20 µm. The graph depicts the quantification of the MFI of MitoSOX. Data are presented as mean ± SEM (n = 3, 15 random fields per condition). ***P < .001, *Vec* versus *ATF4/EGFP*.

Our data indicate that expression of ATF4- and/or Nrf2-dependent antioxidant (*Hmox1/Ho-1*) and GSH-synthesizing (*Cth*, *Gclc*, *Gclm*, *Gsr*, *Slc3a2*, and *Slc7a11*) genes was dysregulated in *A/A* cells during ER stress (Figs. 1C, 1D, S2C, and S2D). Therefore, we examined whether ATF4 OE restores expression of these genes in *A/A* MEFs. To this end, *A/A* MEFs were infected with *Ad-Vector* or *Ad-ATF4/EGFP* and then treated with or without Tm. The mRNA levels of most examined genes (*Nrf2*, *Ho-1*, *Cth*, *Gclc*, *Gsr*, *Slc3a2*, and *Slc7a11*) were significantly higher in *Ad-ATF4-/EGFP-*infected cells than in *Ad-Vector-*infected cells regardless of Tm treatment (Fig. 2A). The mRNA levels of all examined genes, excluding *Gclm*, were further increased by Tm treatment (Fig. 2A). Similarly, the protein levels of HO-1, CTH, GSR, and SLC7A11 were significantly higher in *Ad-ATF4-/EGFP-*infected cells than in *Ad-Vector-*infected cells, regardless of Tm treatment, but were not further increased by Tm treatment (Fig. 2B). ATF4 OE increased levels of mRNAs (*Cth*, *Gclc*, *Gsr*, *Slc3a2*, and *Slc7a11*) and proteins (CTH, GSR, and SLC7A11) related to GSH synthesis; therefore, we assessed the levels of GSH and GSSG in total cellular and mitochondria-enriched fractions of wild-type eIF2α- or ATF4-overexpressing *A/A* MEFs treated with and without Tm (Fig. 2C). Consistent with gene expression analyses (Fig. 2A and B), ATF4 OE strongly restricted changes of cellular and mitochondrial GSH and GSSG levels in Tm-treated *A/A* MEFs, although its effects were weaker than those of wild-type eIF2α OE (Fig. 2C). In addition, measurement of mitochondrial GSH levels in living *A/A* MEFs overexpressing wild-type eIF2α (Flag-eIF2α/iRFP713-Nuc) or ATF4 (HA-ATF4/iRFP713-Nuc) using MitoFreSHtracer confirmed that the eIF2α phosphorylation-ATF4 pathway maintained mitochondrial GSH levels during ER stress (Figs. 2D and S5A-C). Finally, microscopic observations of intracellular and mitochondrial ROS revealed that ATF4 OE efficiently prevented cellular (Fig. 2E) and mitochondrial (Fig. 2F) ROS accumulation in Tm-treated *A/A* MEFs. Our results indicate that the eIF2α phosphorylation-ATF4 pathway is required for proper expression of antioxidant genes, including GSH-synthesizing genes and thereby ameliorates impairment of cellular and mitochondrial redox homeostasis (especially GSH homeostasis) during ER stress.

### eIF2α Phosphorylation Prevents the Exacerbation of NADPH Depletion by Maintaining Optimal Expression of NADPH-Producing Genes During ER Stress

**Fig. 3:**
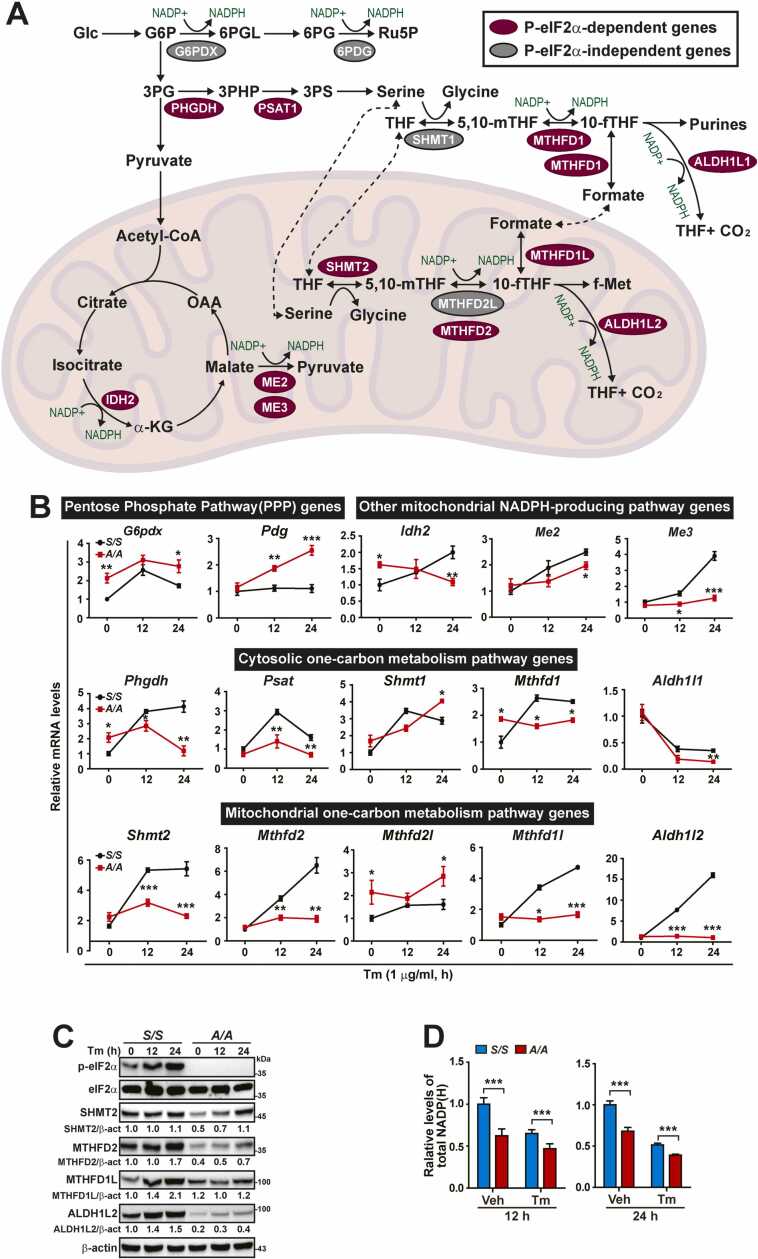
eIF2α phosphorylation deficiency dysregulates the expression of NADPH-producing genes, thereby exacerbating NADPH depletion in *A/A* MEFs during ER stress. (A) Schematic diagram of NADPH-generating pathways. (B) Quantitative RT-PCR analysis of the mRNA levels of pentose phosphate pathway (PPP) genes, cytosolic 1-carbon metabolism pathway gene, mitochondrial 1-carbon metabolism pathway genes, and other mitochondrial NADPH-producing pathway genes in *S/S* and *A/A* MEFs treated with Tm for the indicated durations. Data are presented as mean ± SEM (n = 3). *P < .05, **P < .01, and ***P < .001, *S/S* versus *A/A* at each time point. (C) WB analysis of mitochondrial 1-carbon metabolism pathway proteins in lysates of *S/S* and *A/A* MEFs treated with Tm for the indicated durations. Protein levels normalized by β-act levels are shown below the panels. (E) Quantification of total NADP(H) levels in *S/S* and *A/A* MEFs treated with Veh or Tm for 12 or 24 h. Data are presented as mean ± SEM (n = 3). ***P < .001, *S/S* versus *A/A*.

NADPH is crucial for reducing cystine, oxidized glutathione (GSSG), and oxidized thioredoxin through specific enzymes. These reduction processes protect cells from oxidative stress and help maintain redox balance (Hine and Mitchell, 2012, Ribas et al., 2014). Furthermore, ATF4, a target of eIF2α phosphorylation, is known to regulate the expression of several genes related to the PPP and serine-driven 1-carbon metabolism involved in NADPH production (Wang et al., 2022, Xia et al., 2019). Therefore, we investigated the expression changes of NADPH-producing genes (Fig. 3A) in *S/S* and *A/A* MEFs under ER stress conditions. The mRNA levels of most examined genes (*Phgdh*, *Psat*, *Mthfd1*, *Aldh1l1*, *shmt2*, *Mthfd2*, *Mthfd1l*, *Aldh1l2*, *Idh2*, *Me2*, and *Me3*) were lower in *A/A* MEFs compared with *S/S* MEFs at a late time point (24 h) or both time points (12 and 24 h) (Fig. 3B). Similarly, in *A/A*^*Hep*^ cells, the mRNA levels of the same 11 genes plus *shmt1* were lower than those in *S/S*^*Hep*^ cells during ER stress (Fig. S6). Among the examined genes, all mitochondrial NADPH-producing genes, except for the *Mthfd2l* gene, showed significantly reduced expression in *A/A* cells compared with *S/S* cells under ER stress conditions (Figs. 3B and S6). Additionally, a deficiency in eIF2α phosphorylation decreased the protein levels of mitochondrial 1-carbon pathway genes, regardless of ER stress for SHMT2, MTHFD2, and ALDH1L2, or specifically during ER stress for MTHFD1L (Fig. 3C). Consistently, Tm treatments reduced total NADP(H) levels more significantly in *A/A* MEFs than in *S/S* MEFs, even though these levels decreased in both *S/S* and *A/A* MEFs (Fig. 3D). Together, these results suggest that eIF2α phosphorylation is necessary for the optimal expression of NADPH-producing genes and for maintaining NADPH homeostasis during ER stress.

**Fig. 4:**
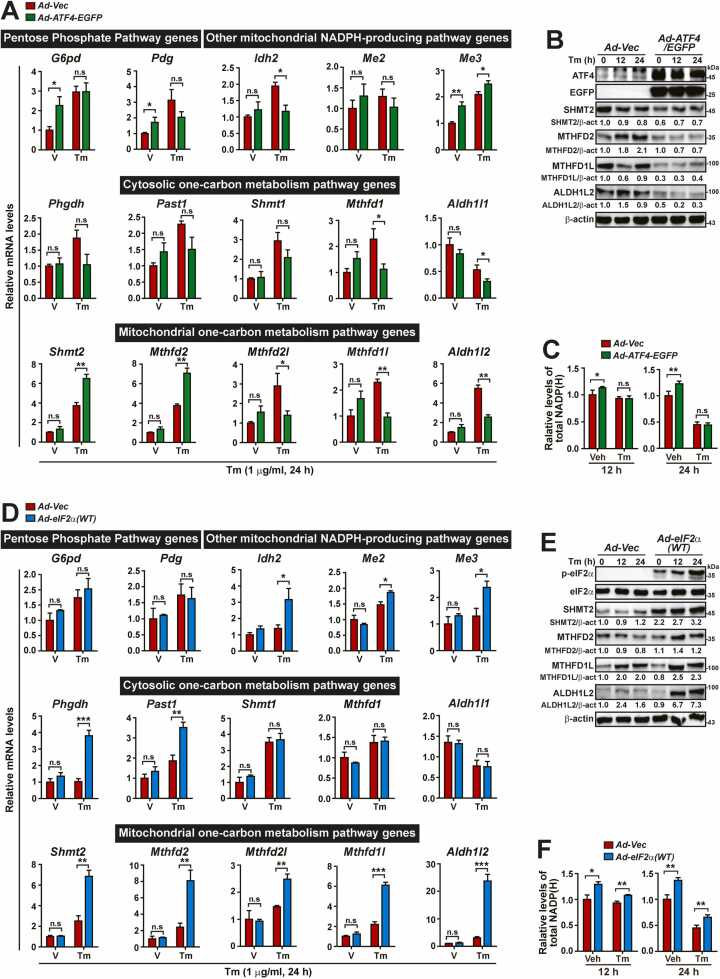
Wild-type eIF2α OE, but not ATF4 OE, restores the expression of NADPH-producing genes, thereby suppressing the exacerbation of NADPH depletion in *A/A* MEFs during ER stress. (A) Quantitative RT-PCR analysis of the mRNA levels of PPP genes, cytosolic 1-carbon metabolism pathway gene, mitochondrial 1-carbon metabolism pathway genes, and other mitochondrial NADPH-producing pathway genes in Vec- or ATF4-/EGFP-overexpressing *A/A* MEFs treated with vehicle (V) or Tm for 24 h. Data are presented as mean ± SEM (n = 3). *P < .05 and **P < .01, *Vec* versus *ATF4/EGFP*. (B) WB analysis of mitochondrial 1-carbon metabolism pathway proteins in lysates of Vec- or ATF4-/EGFP-overexpressing *A/A* MEFs treated with Tm for the indicated durations. Protein levels normalized by β-act levels are shown below the panels. (C) Quantification of total NADP(H) levels in Vec- or ATF4/EGFP-MEFs treated with Veh or Tm for 12 or 24 h. Data are presented as mean ± SEM (n = 3). *P < .05 and **P < .01, *Vec* versus *ATF4/EGFP*. (D) Quantitative RT-PCR analysis of the mRNA levels of PPP genes, cytosolic 1-carbon metabolism pathway gene, mitochondrial 1-carbon metabolism pathway genes, and other mitochondrial NADPH-producing pathway genes Vec- or eIF2α(WT)-overexpressing *A/A* MEFs treated with vehicle (V) or Tm for 24 h. Data are presented as mean ± SEM (n = 3). *P < .05, **P < .01, and ***P < .001, *Vec* versus *eIF2α(WT)*. (E) WB analysis of mitochondrial 1-carbon metabolism pathway proteins in lysates of Vec- or eIF2α(WT)-overexpressing *A/A* MEFs treated with Tm for the indicated durations. Protein levels normalized by β-act levels are shown below the panels. (F) Quantification of total NADP(H) levels in Vec- or eIF2α(WT)-MEFs treated with Veh or Tm for 12 or 24 h. Data are presented as mean ± SEM (n = 3). *P < .05 and **P < .01, *Vec* versus *eIF2α(WT)*.

ATF4 OE rescued the dysregulation of GSH homeostasis in *A/A* MEFs (Figs. 2 and S5), as well as mitochondrial dynamics and mtDNA replication in *Atf4*^*-/-*^ and *A/A* MEFs during ER stress (Le et al., 2025). Therefore, we investigated whether it can prevent the dysregulated expression of genes related to NADPH production in eIF2α phosphorylation-deficient cells during ER stress. Surprisingly, among the examined genes affected by eIF2α phosphorylation deficiency, the transcript levels of most genes, except for *Shmt2*, *Mthfd2*, and *Me3*, remained unchanged or were even oppositely decreased by ATF4 OE during ER stress (Fig. 4A). Furthermore, ATF4 OE in *A/A* MEFs further reduced the protein levels of mitochondrial 1-carbon pathway genes, regardless of ER stress for SHMT2, MTHFD1L, and ALDH1L2, or specifically during ER stress for MTHFD2 (Fig. 4B). Therefore, ATF4 OE did not prevent the exacerbation of NADPH depletion in *A/A* MEFs during ER stress, although it slightly increased the total NADP(H) level under normal conditions (Fig. 4C). Together, these results suggest that the impairment of NADPH homeostasis is not merely due to a lack of ATF4 expression in eIF2α phosphorylation-deficient cells during ER stress.

Consequently, we deemed it necessary to investigate whether the dysregulated expression of NADPH-producing genes in *A/A* MEFs could be rescued by wild-type eIF2α OE during ER stress. As expected, OE of wild-type eIF2α restored the mRNA levels of most decreased genes (*Phgdh*, *Psat*, *Shmt2*, *Mthfd2*, *Mthfd1l*, *Aldh1l2*, *Idh2*, *Me2*, and *Me3*) as well as the protein levels of SHMT2, MTHFD2, MTHFD1L, and ALDH1L2 in *A/A* MEFs during ER stress (Fig. 4D and E). Consistently, wild-type eIF2α OE increased total NADP(H) levels in *A/A* MEFs under both normal and ER stress conditions (Fig. 4F). Therefore, our results indicate that there is no issue with the biological identity of our eIF2α phosphorylation-deficient cells.

### eIF2α Phosphorylation Deficiency Exacerbates Ferroptosis During ER Stress

**Fig. 5:**
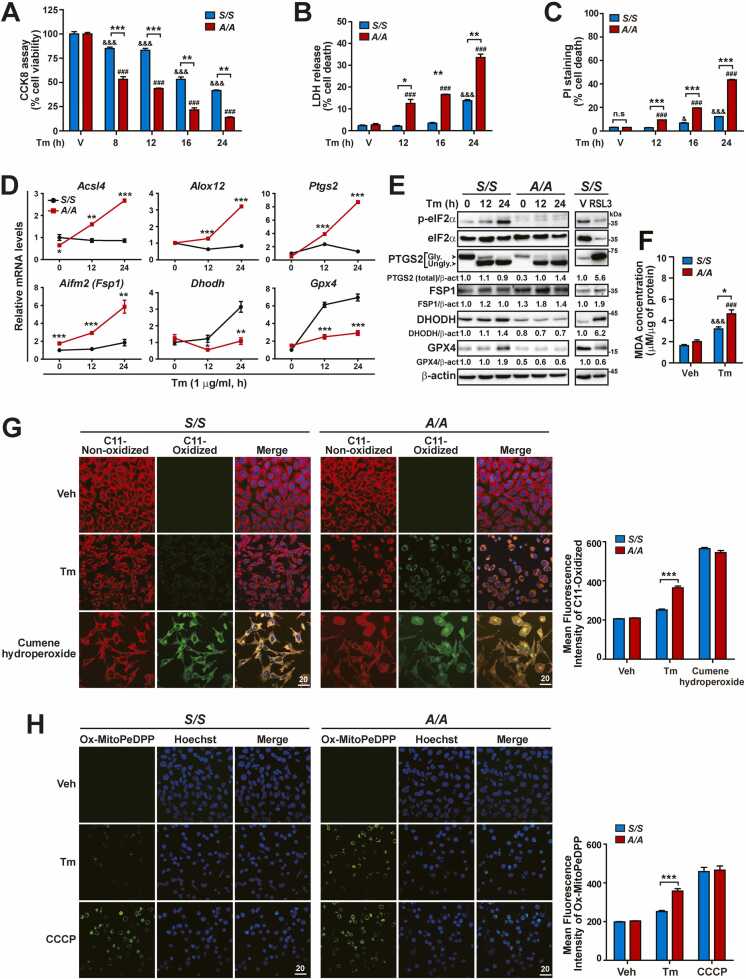
eIF2α phosphorylation is required to suppress ferroptosis during ER stress. (A-C) Cell viability measurements by the CCK-8 assay (A), cell death measurements by the LDH release assay (B), and PI staining (C, flow cytometric analysis) in *S/S* and *A/A* MEFs treated with Veh (24 h) or Tm for the indicated durations. Data are presented as mean ± SEM (n = 3). *P < .05, **P < .01, and ***P < .001, *S/S* versus *A/A*; ^&^*P* < .05 and ^&&&^*P* < .001, Veh (V) versus each time point in *S/S*; ^###^*P* < .001, Veh (V) versus each time point in *A/A*. (D) Quantitative RT-PCR analysis of mRNA levels of ferroptosis-activating (*Acsl4*, *Alox12*, and *Ptgs2*) and ferroptosis-inhibiting (*Aifm2*, *Dhodh*, and *Gpx4*) genes in *S/S* and *A/A* MEFs treated with Tm for the indicated durations. Data are presented as mean ± SEM (n = 3). *P < .05, **P < .01, and ***P < .001, *S/S* versus *A/A*. (E) WB analysis of ferroptosis-related proteins in lysates of *S/S* and *A/A* MEFs treated with Tm for the indicated durations. To induce ferroptosis, *S/S* MEFs were treated with Veh (V) or RSL3 (5 µM) for 6 h. PTGS2 was deglycosylated by Tm treatment. Gly, glycosylated PTGS2; Ungly, unglycosylated PTGS2. Protein levels normalized by β-act levels are shown below the panels. (F) Measurement of MDA levels in *S/S* and *A/A* MEFs treated with Veh or Tm for 24 h. Data are presented as mean ± SEM (n = 3). *P < .05, *S/S* versus *A/A*; ^&&&^*P* < .001, Veh versus Tm in *S/S*; ^###^*P* < .001, Veh versus Tm in *A/A*. (G) Representative BODIPY 581/591 C11 staining images of *S/S* and *A/A* MEFs. Cells were treated with Veh (24 h), Tm (24 h), or cumene hydroperoxide (100 µM, 2 h) and stained with the intracellular lipid peroxidation probe BODIPY 581/591 C11 (red and green) and Hoechst 33258 (blue) for the last 30 min. Cumene hydroperoxide was used as a lipid peroxidation-inducing agent. Scale bar: 20 µm. The graph depicts the quantification of the MFI of C11-Oxidized. Data are presented as mean ± SEM (n = 3, 15 random fields per condition). ***P < .001, *S/S* versus *A/A*. (H) Representative Ox-MitoPeDPP staining images of *S/S* and *A/A* MEFs. Cells were treated with Veh (24 h), Tm (24 h), or carbonyl cyanide-m-chlorophenyl hydrazone (CCCP, 4 µM, 4 h) and stained with the mitochondrial lipid peroxidation probe MitoPeDPP (green) and Hoechst 33258 (blue) for the last 30 min. Scale bar: 20 µm. The graph depicts the quantification of the MFI of Ox-MitoPeDPP. Data are presented as mean ± SEM (n = 3, 15 random fields per condition). ***P < .001, *S/S* versus *A/A*.

Upon Tm treatment, *A/A* cells displayed intracellular and mitochondrial GSH depletion and ROS accumulation, which were expected to cause cell death; therefore, we compared cell viability and death between Tm-treated *S/S* and *A/A* MEFs. As reported previously (Choi et al., 2017), microscopic observation and CCK-8, LDH release, and PI/Hoechst staining assays revealed that *A/A* MEFs exhibited decreased viability and increased death compared with *S/S* MEFs at all time points after Tm and Tg treatments (Figs. 5A-C, S7A, and S8A-C). This indicates that deficiency of eIF2α phosphorylation makes cells more sensitive to Tm and Tg treatments. Furthermore, the active form of caspase 3 (cleaved caspase 3) was observed in both *S/S* and *A/A* cells at late time points (16 and 24 h) of Tm treatment (Fig. S2E and S7B). However, the levels of cleaved caspase 3 and cleaved PARP, a target protein of caspase 3, were higher in *A/A* cells than in *S/S* cells upon Tm treatment (Fig. S2E and S7B). These results indicate that eIF2α phosphorylation deficiency aggravates caspase-mediated apoptosis, which may be one of the major cell death pathways responsible for the increased death of *A/A* cells during ER stress. Next, we performed time-dependent Annexin V and 7-AAD staining assays to carefully analyze cell death pathways induced in Tm-treated *S/S* and *A/A* MEFs over 24 h. Tm treatment increased both Annexin V-positive cells (Q4, indicating apoptosis) and 7-AAD-positive cells (Q2, indicating other forms of cell death involving the loss of plasma membrane integrity) among both *S/S* and *A/A* MEFs (Fig. S7C). However, with longer Tm treatment durations, the increase of 7-AAD-positive cells (Q2) was more pronounced among *A/A* MEFs, while the increase of Annexin V-positive cells (Q4) was more pronounced among *S/S* MEFs (Fig. S7C). This indicates that eIF2α phosphorylation is also required to suppress other forms of cell death involving the loss of plasma membrane integrity under ER stress conditions. Furthermore, we performed Annexin V and 7-AAD staining assays in Tm-treated *A/A* MEFs with and without Z-VAD-FMK (Z-VAD, a cell-permeant pan-caspase inhibitor of apoptosis) (Fig. S7D). As expected, Z-VAD treatment significantly reduced the number of Annexin V and 7-AAD-double-positive cells (Q3) but, rather intriguingly, increased the number of 7-AAD-positive cells (Q2) among Tm-treated *A/A* MEFs. Together, our data indicate that ER stress in eIF2α phosphorylation-deficient cells induces cell death, which cannot be prevented by an apoptosis inhibitor, involving the loss of plasma membrane integrity at earlier time points (8 and 12 h, Fig. S7C) before the activation of apoptosis.

Among several cell death pathways (Iurlaro and Munoz-Pinedo, 2016, Zhang et al., 2022b) that may be responsible for death of *A/A* cells under ER stress conditions, we speculated that Tm treatment of *A/A* cells leads to ferroptosis, which is a newly discovered type of nonapoptotic cell death triggered by conditions that inhibit GSH biosynthesis or the GSH-dependent antioxidant enzyme GPX4 (Cao and Dixon, 2016, Jiang et al., 2021, Stockwell, 2022). To provide evidence that Tm-treated *A/A* cells undergo ferroptotic death, the mRNA and protein expression levels of ferroptosis-activating (*Acsl4*, *Alox12*, and *Ptgs2*) and ferroptosis-suppressing (*Aifm2/Fsp1*, *Dhodh*, and *Gpx4*) (Cao and Dixon, 2016, Jiang et al., 2021, Stockwell, 2022) genes in *A/A* cells were compared with those in *S/S* cells. The mRNA expression levels of ferroptosis-activating genes (*Acsl4*, *Alox12*, and *Ptgs2*) were substantially higher in *A/A* cells than in *S/S* cells during ER stress (Figs. 5D and S2F). In addition, the PTGS2 level was drastically increased by Tm treatment in *A/A* cells because it was significantly lower in these cells than in *S/S* cells before Tm treatment (Figs. 5E and S2G). However, the mRNA expression levels of ferroptosis-suppressing genes (*Dhodh* and *Gpx4*) were significantly lower in *A/A* cells than in *S/S* cells, although the levels of *Aifm2/Fsp1* transcripts were higher in *A/A* cells than in *S/S* cells under normal conditions and were further increased by Tm treatment (Figs. 5D and S2F). In *A/A* cells, the protein levels of ferroptosis-suppressing genes (*Dhodh* and *Gpx4*) were lower, while the level of FSP1 protein was higher, compared with *S/S* cells, regardless of ER stress (Fig. 5E and S2G). To evaluate the role of upregulated *Fsp1* gene expression, cell viability assays using iFSP1, an FSP1 inhibitor, were conducted with *A/A* MEFs treated with RSL3, a ferroptosis activator, and Tm, an ER stress inducer (Fig. S9). Increasing iFSP1 concentrations further reduced the viability of *A/A* cells under the Veh-treated condition as well as upon treatment with RSL3 (0.5 and 2.5 µM, Fig. S9A) and Tm (0.1 and 0.5 µg/ml Tm, Fig. S9B), implying that the increased level of FSP1 protects *A/A* cells from a certain degree of ferroptosis under ER stress conditions (up to 0.5 µg/ml Tm). However, cell death was not exacerbated by FSP1 inhibition upon treatment with the highest RSL3 (5 µM, Fig. S9A) and Tm (1 µg/ml, Fig. S9B). This suggests that the elevated level of FSP1 is inadequate to counter the intense ferroptosis induced by 1 µg/ml Tm treatment in GSH-depleted *A/A* cells (Fig. 1E-I), alongside the dysregulated expression of ferroptosis-regulating genes (Figs. 5D, 5E, S2F, and S2G). However, further investigations are required to determine why and how the levels of *Fsp1* mRNA and its protein are increased in *A/A* cells under normal conditions and ER stress conditions. Finally, the levels of 2 specific indicators of ferroptosis, MDA and lipid peroxides from both cellular and mitochondrial sources, were assessed in *S/S* and *A/A* MEFs during ER stress. Consistent with our expectation, MDA levels were significantly higher in *A/A* MEFs than in *S/S* MEFs upon Tm and Tg treatments (Figs. 5F and S8D). BODIPY 581/591 C11 and MitoPeDPP, which are lipid peroxidation probes, were used to measure the levels of intracellular (Figs. 5G and S8E) and mitochondrial (Figs. 5H and S8F) lipid peroxidation, respectively. Similar to the MDA results, the levels of both intracellular (C11-Oxidized) and mitochondrial (Ox-MitoPeDPP) lipid peroxidation were notably higher in *A/A* MEFs than in *S/S* MEFs upon Tm and Tg treatments. However, no significant differences were observed in cumene hydroperoxide-mediated intracellular lipid peroxidation or CCCP-mediated mitochondrial lipid peroxidation between *S/S* and *A/A* cells (Figs. 5G and H). Collectively, there was a depletion of cellular and mitochondrial GSH (Figs. 1E-I, S1C, and S1D) due to the dysregulated expression of genes related to GSH synthesis (*Cth*, *Gclc*, *Gsr*, *Slc3a2*, and *Slc7a11*) in *A/A* cells during ER stress conditions (Figs. 1C-I, S2C, and S2D). Furthermore, *A/A* cells exhibited exacerbated NADPH depletion due to the dysregulated expression of genes related to NADPH production (*Phgdh*, *Psat*, *Shmt2*, *Mthfd2*, *Mthfd1l*, *Aldh1l2*, *Idh2*, *Me2*, and *Me3*) during ER stress conditions (Figs. 3 and 4). Additionally, there was dysregulated expression of ferroptosis-suppressing (*Aifm2/Fsp1*, *Dhodh*, and *Gpx4*) and ferroptosis-activating (*Acsl4*, *Alox12*, and *Ptgs2*) genes (Figs. 5D, 5E, S2F, and S2G), accompanied by increased accumulation of oxidized lipids in Tm- and Tg-treated *A/A* cells (Figs. 5G, 5H, S8E, and S8F). All of these observations are related to ferroptosis. These results strongly indicate that eIF2α phosphorylation deficiency leads to increased ferroptosis during ER stress.

### GSH Replenishment Efficiently Suppresses Ferroptosis of *A/A* Cells During ER Stress

**Fig. 6:**
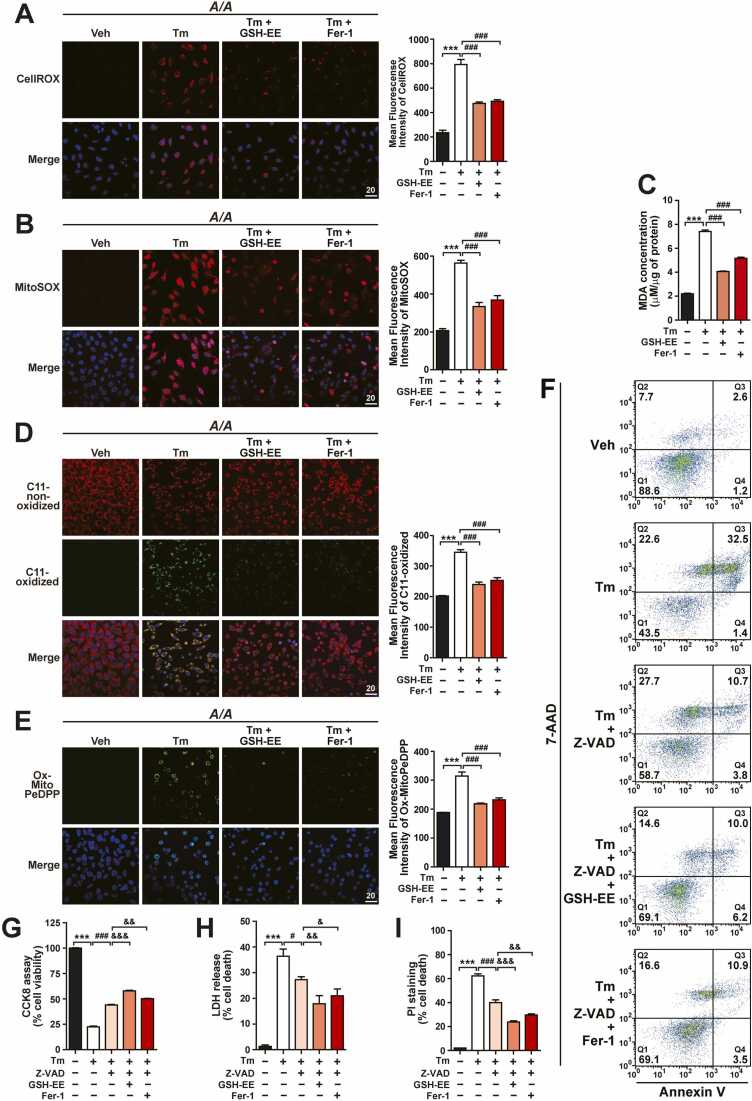
GSH supplementation suppresses lipid peroxidation and subsequently ferroptosis in Tm-treated *A/A* cells. (A and B) Representative CellROX and MitoSOX staining images of *A/A* MEFs. Cells were treated with Veh, Tm, Tm plus GSH-EE (0.75 µM), or Tm plus Fer-1 (5 µM) for 24 h and stained with either CellROX Deep Red (red) and Hoechst 33258 (blue) or MitoSOX Red (red) and Hoechst 33258 (blue) for the last 30 min. Before treatment with Tm or Tm plus GSH-EE or Fer-1, cells were pretreated with Veh (-), GSH-EE, or Fer-1 for 1 h. Scale bar: 20 µm. The graphs depict the quantification of the MFI of CellROX or MitoSOX. Data are presented as mean ± SEM (n = 3, 15 random fields per condition). ***P < .001, Veh versus Tm; ^###^*P* < .001, Tm versus either Tm plus GSH-EE or Tm plus Fer-1. (C) Measurement of MDA levels in *A/A* MEFs treated with Veh, Tm, Tm plus GSH-EE, or Tm plus Fer-1 for 24 h. Before treatment with Tm or Tm plus GSH-EE or Fer-1, cells were pretreated with Veh (-), GSH-EE, or Fer-1 for 1 h. Data are presented as mean ± SEM (n = 3). ***P < .001, Veh versus Tm; ^###^*P* < .001, Tm versus either Tm plus GSH-EE or Tm plus Fer-1. (D and E) Representative C11-Oxidized and Ox-MitoPeDPP staining images of *A/A* MEFs. Cells were treated with Veh, Tm, Tm plus GSH-EE (0.75 µM), or Tm plus Fer-1 (5 µM) for 24 h and stained with either BODIPY 581/591 C11 (red and green) and Hoechst 33258 (blue) or MitoPeDPP (green) and Hoechst 33258 (blue) for the last 30 min. Before treatment with Tm or Tm plus GSH-EE or Fer-1, cells were pretreated with Veh (-), GSH-EE, or Fer-1 for 1 h. Scale bar: 20 µm. The graphs depict the quantification of the MFI of C11-Oxidized or Ox-MitoPeDPP. Data are presented as mean ± SEM (n = 3, 15 random fields per condition). ***P < .001, Veh versus Tm; ^###^*P* < .001, Tm versus either Tm plus GSH-EE or Tm plus Fer-1. (F) Representative flow cytometric analysis images of Annexin V- and 7-AAD-stained *A/A* MEFs treated with Veh, Tm, Tm plus GSH-EE, or Tm plus Fer-1 for 24 h. Before treatment with Tm or Tm plus the indicated chemicals, cells were pretreated with Veh (-), Z-VAD, Z-VAD plus GSH-EE, or Z-VAD plus Fer-1 for 1 h. The values shown represent the means (%) (n = 3). (G-I) Cell viability measurements by the CCK-8 assay (G) and cell death measurements by the LDH release assay (H) and PI staining (I, flow cytometric analysis) in *A/A* MEFs treated with Veh, Tm, Tm plus Z-VAD, Tm plus GSH-EE, or Tm plus Fer-1 for 24 h. Before treatment with Tm or Tm plus the indicated chemicals, cells were pretreated with Veh (-), Z-VAD, Z-VAD plus GSH-EE, or Z-VAD plus Fer-1 for 1 h. Data are presented as mean ± SEM (n = 3). ***P < .001, Veh versus Tm; ^#^*P* < .05 and ^###^*P* < .001, Tm versus Tm plus Z-VAD; ^&^*P* < .05, ^&&^*P* < .01, and ^&&&^*P* < .001, Tm plus Z-VAD versus either Tm plus Z-VAD plus GSH-EE or Tm plus Z-VAD plus Fer-1.

GSH depletion, which is closely associated with ferroptosis (Cao and Dixon, 2016, Jiang et al., 2021, Stockwell, 2022), was observed in *A/A* cells during ER stress (Figs. 1E-I, S1C, and S1D). Therefore, we examined whether GSH supplementation restrains the ferroptotic death of *A/A* cells during ER stress. Surprisingly, glutathione ethyl ester (GSH-EE, a cell-permeable derivative of GSH) substantially suppressed cellular (Fig. 6A) and even mitochondrial (Fig. 6B) ROS accumulation in Tm-treated *A/A* MEFs. In addition, the ROS-scavenging ability of GSH-EE was stronger than that of Fer-1 (a potent inhibitor of ferroptosis) (0.75 µM GSH-EE vs 5 µM Fer-1, Fig. 6A and B). Consistent with the decreased ROS levels, GSH-EE treatment significantly reduced the levels of MDA (Fig. 6C), C11-Oxidized (Fig. 6D), and Ox-MitoPeDPP (Fig. 6E) in *A/A* MEFs under Tm-treated conditions, indicating that GSH depletion is a critical factor for ferroptosis induction in *A/A* cells during ER stress. The inhibitory effect of GSH-EE on lipid peroxidation was stronger than that of Fer-1 (0.75 µM GSH-EE vs 5 µM Fer-1, Fig. 6C-E). Consistent with the results presented in Figure 6A-E, the CCK-8, LDH release, and PI/Hoechst staining assays, along with microscopic observations, revealed that GSH supplementation increased viability (Fig. S10A and D) and reduced death (Fig. S10B and C) of *A/A* MEFs under Tm-treated conditions. In addition, the effects of GSH supplementation were stronger than those of Fer-1 treatment (Fig. S10A-D). However, GSH and Fer-1 treatments did not reduce caspase-3 activation and PARP cleavage (Fig. S10E), suggesting that GSH depletion-mediated ferroptosis is an independent cell death pathway distinct from apoptosis in Tm-treated *A/A* MEFs. We further investigated how strongly pharmacological inhibition of both apoptosis and ferroptosis suppresses Tm-induced death of *A/A* cells. Microscopic analysis revealed that co-treatment with Z-VAD and either GSH-EE or Fer-1 more effectively prevented cell death than treatment with Z-VAD alone (Fig. S10F). Next, Annexin V and 7-AAD staining assays revealed that treatment with Z-VAD alone significantly reduced the number of Annexin V and 7-AAD double-positive cells (Q3) but increased the number of 7-AAD-positive cells (Q2) (Appendix A, Fig. 6). However, co-treatment with Z-VAD and either GSH-EE or Fer-1 substantially decreased the numbers of Annexin V and 7-AAD double-positive cells (Q3) and 7-AAD-positive cells (Q2) among Tm-treated *A/A* MEFs (Fig. 6F), indicating that nearly half of 7-AAD-positive cells (Q2) exhibiting loss of plasma membrane integrity were generated by ferroptotic cell death (26.0%, Q2 of Tm plus Z-VAD vs 13.7%, Q2 of Tm plus Z-VAD plus GSH-EE). In addition, the CCK-8, LDH release, and PI/Hoechst staining assays revealed that co-treatment with Z-VAD and either GSH-EE or Fer-1 additively increased viability (Fig. 6G) and additively suppressed death (Fig. 6H and I) of Tm-treated *A/A* MEFs. Together, our findings indicate that GSH depletion plays a crucial role in inducing ferroptosis in cells deficient in eIF2α phosphorylation during ER stress, forming a cell death pathway distinct from apoptosis.

### ATF4 OE Mitigates Ferroptosis as Well as Apoptosis of *A/A* Cells During ER Stress

**Fig. 7:**
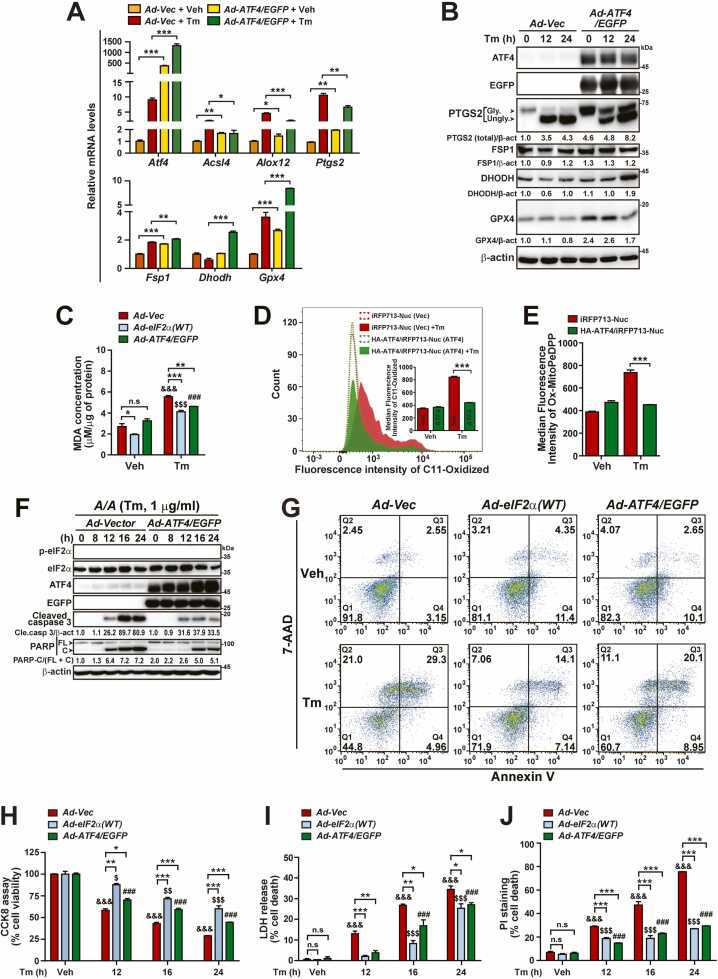
ATF4 OE modulates the expression of ferroptosis-related genes and mitigates ferroptosis as well as apoptosis in *A/A* cells. (A) Quantitative RT-PCR analysis of mRNA levels of ferroptosis-activating (*Acsl4*, *Alox12*, and *Ptgs2*) and ferroptosis-inhibiting (*Aifm2*, *Dhodh*, and *Gpx4*) genes in Vec- or ATF4-/EGFP-overexpressing *A/A* MEFs treated with Veh or Tm for 24 h. Data are presented as mean ± SEM (n = 3). *P < .05, **P < .01, and ***P < .001, *Vec* versus *ATF4/EGFP*. (B) WB analysis of ferroptosis-related proteins in lysates of Vec- or ATF4-/EGFP-overexpressing *A/A* MEFs treated with Tm for the indicated durations. Protein levels normalized by β-act levels are shown below the panels. (C) Measurement of MDA levels in Vec-, eIF2α(WT), or ATF4-/EGFP-overexpressing *A/A* MEFs treated with Veh or Tm for 24 h. Data are presented as mean ± SEM (n = 3). *P < .05 and ***P < .001, *Vec* versus *eIF2α(WT)* or *ATF4/EGFP*; ^&&&^*P* < .001, Veh versus Tm in *Vec*; ^$$$^*P* < .001, Veh versus Tm in *eIF2α(WT)*; ^###^*P* < .001, Veh versus Tm in *ATF4/EGFP*. (D) A representative histogram of the fluorescence intensity of C11-oxidized with an inset graph showing the median fluorescence intensity in *A/A* MEFs overexpressing iRFP713-Nuc (Vec) or HA-ATF4/iRFP713-Nuc (ATF4). Transfected cells were treated with Veh or Tm for 24 h and stained with BODIPY 581/591 C11 for the last 30 min. The fluorescence intensity of C11-oxidized positive cells was measured among cells gated with far-red fluorescence using flow cytometry. (E) A graph showing the median fluorescence intensity of Ox-MitoPeDPP in iRFP713-Nuc- or HA-ATF4/iRFP713-Nuc-overexpressing *A/A* MEFs treated with Veh or Tm for 24 h. The fluorescence intensity of Ox-MitoPeDPP-positive cells was measured among cells gated with far-red fluorescence using flow cytometry. Data are presented as mean ± SEM (n = 3). ***P < .001, *iRFP713-Nuc* versus *HA-ATF4/iRFP713-Nuc*. (F) WB analysis of cleaved caspase 3 and PARP in lysates of Vec- or ATF4-/EGFP-overexpressing *A/A* MEFs treated with Tm for the indicated durations. Protein levels normalized by β-act levels are shown below the panels. (G) Representative flow cytometric analysis images of Annexin V- and 7-AAD-stained Vec-, eIF2α(WT)-, or ATF4-overexpressing *A/A* MEFs treated with Veh or Tm for 24 h. The values shown represent the means (%) (n = 3). (H-J) Cell viability measurements by the CCK-8 assay (H), cell death measurements by the LDH release assay (I), and PI staining (J) in Vec-, eIF2α(WT)-, or ATF4-overexpressing *A/A* MEFs treated with Veh (24 h) or Tm for the indicated durations. Data are presented as mean ± SEM (n = 3). *P < .05, **P < .01, and ***P < .001, *Vec* versus *eIF2α-WT* or *ATF4/EGFP*; ^&&&^*P* < .001, Veh versus each time point in *Vec*; ^$^*P* < .05, ^$$^*P* < .01, and ^$$$^*P* < .001, Veh versus each time point in *eIF2α(WT)*; ^###^*P* < .001, Veh versus each time point in *ATF4/EGFP*; n.s, not significant.

ATF4 OE enhanced expression of *Nfe2l2/Nrf2* and multiple genes (*Cth*, *Gclc*, *Gsr*, *Slc3a2*, and *Slc7a11*) related to GSH synthesis (Fig. 2A and B) and thereby restored intracellular and mitochondrial GSH contents (Figs. 2C, 2D, and S5) in *A/A* MEFs under ER stress conditions. In addition, it was reported that Nrf2 is an important transcriptional regulator of antiferroptotic genes involved in iron metabolism, ROS detoxification, and GSH synthesis (Dai et al., 2020, Dodson et al., 2019). Therefore, we investigated whether ATF4 OE changes the expression patterns of ferroptosis-activating (*Acsl4*, *Alox12*, and *Ptgs2*) and ferroptosis-suppressing (*Aifm2/Fsp1*, *Dhodh*, and *Gpx4*) genes and thereby prevents ferroptosis of *A/A* MEFs during ER stress. As expected, ATF4 OE decreased the mRNA levels of ferroptosis-activating genes (*Acsl4*, *Alox12*, and *Ptgs2*) but increased the mRNA levels of ferroptosis-suppressing genes (*Fsp1*, *Dhodh,* and *Gpx4*) in Tm-treated *A/A* MEFs (Fig. 7A). In addition, ATF4 OE reduced Tm-mediated induction of PTGS2 protein from ∼4-fold (0 h vs 24 h in *Ad-Vec*) to ∼1.8-fold (0 h vs 24 h in *Ad-ATF4/EGFP*) because the PTGS2 protein level was considerably upregulated in ATF4-overexpressing *A/A* MEFs without Tm treatment (Fig. 7B). More importantly, ATF4 OE increased expression of the ferroptosis-suppressing proteins DHODH and GPX4 in *A/A* MEFs during ER stress (Fig. 7B), although the FSP1 protein level was slightly increased by ATF4 OE but was not notably changed by Tm treatment (Fig. 7B). Therefore, ATF4 OE reduced the level of MDA, a final product of lipid peroxidation, in Tm-treated *A/A* MEFs. ATF4 OE suppressed the MDA level similar to wild-type eIF2α OE in Tm-treated *A/A* MEFs, although OE of wild-type eIF2α, but not of ATF4, reduced the MDA level in *A/A* MEFs before Tm treatment (Fig. 7C). In addition, measurements of both C11-Oxidized- and Ox-MitoPeDPP-positive cells among living *A/A* MEFs overexpressing vector (iRFP713-Nuc) or ATF4 (HA-ATF4/iRFP713-Nuc) using a flow cytometer confirmed that ATF4 OE reduced both intracellular (C11-Oxidized, Fig. 7D) and mitochondrial (Ox-MitoPeDPP, Fig. 7E) lipid peroxidation to almost basal levels. Thus, ATF4 OE significantly reduced the levels of 3 specific indicators of ferroptosis that were increased by eIF2α phosphorylation deficiency during ER stress. Finally, to verify whether ATF4 OE increases viability and decreases the death of Tm-treated *A/A* MEFs, we performed the same assays as shown in Figure 6F-I and S10E. Contrary to the results obtained upon GSH-EE treatment (Fig. S10E), ATF4 OE reduced caspase-3 activation and PARP cleavage in Tm-treated *A/A* MEFs (Fig. 7F), suggesting that ATF4 reduces apoptosis upregulated by eIF2α phosphorylation deficiency during ER stress. Next, Annexin V and 7-AAD staining assays revealed that ATF4 OE decreased the numbers of Annexin V-positive cells (Q3) and 7-AAD-positive cells (Q2), although the effects of wild-type eIF2α OE were stronger than those of ATF4 OE (Fig. 7G). Together, these data indicate that ATF4 OE can reduce both apoptosis and ferroptosis in Tm-treated *A/A* cells. Consistent with the results presented in Figure 7A-G, the CCK-8, LDH release, and PI/Hoechst staining assays revealed that OE of wild-type eIF2α and ATF4 increased viability (Fig. 7H) and reduced death (Fig. 7I and J) of *A/A* MEFs at all time points after Tm treatment, although the effects of wild-type eIF2α OE seemed slightly stronger than those of ATF4 OE (Fig. 7H and I). Thus, the eIF2α phosphorylation-ATF4 pathway mitigates ferroptosis as well as apoptosis during ER stress.

## DISCUSSION

The present study demonstrated that eIF2α phosphorylation plays an essential role in the preservation of redox homeostasis (especially GSH and NADPH homeostasis) by mediating transcriptional reprogramming during ER stress. Consequently, eIF2α phosphorylation restrains not only apoptosis but also ferroptosis during ER stress (see Graphical abstract). We found that ATF4 OE, which was significantly downregulated in *A/A* cells during ER stress, ameliorated the disruption of redox homeostasis (reflected in cellular and mitochondrial ROS and GSH levels) by promoting the expression of multiple genes involved in GSH synthesis, although it did not prevent exacerbated NADPH depletion. Collectively, our results reveal how eIF2α phosphorylation induces transcriptional reprogramming via its downstream TF ATF4 and unidentified eIF2α phosphorylation-dependent target(s), allowing cells to overcome or adapt to ER stress.

ER stress can generate ROS by cellular processes, including oxidative protein folding and mitochondrial respiration (Ong and Logue, 2023, Santos et al., 2009). In addition, ATF4 is a critical TF for resistance to oxidative stress during ER stress because it induces transcriptional expression of genes involved in redox homeostasis, including GSH synthesis (such as *Ho-1*, *Cth, Slc3a2*, and *Slc7a11*) (Dickhout et al., 2012, Harding et al., 2003, Lewerenz and Maher, 2009). Therefore, the deficiency of ATF4 expression may be responsible for the increased sensitivity of *A/A* cells to oxidative stress under ER stress conditions (Back et al., 2009, Choi et al., 2017, Dickhout et al., 2012, Harding et al., 2003, Lewerenz and Maher, 2009). As expected, ATF4 OE increased GSH levels in both the cytosol and mitochondria and inhibited intracellular and mitochondrial ROS accumulation by stimulating transcription of diverse antioxidant genes, including GSH-synthesizing genes in *A/A* cells under ER stress conditions (Figure 2). However, reports from other groups (Kress et al., 2023, Sarcinelli et al., 2020) and our data (Figures 1C, 1D, 2A, 2B, S2C, S2D, S3B, and S4A-G) indicate that Nrf2 is a direct transcriptional target of ATF4 and that ATF4 and Nrf2 cooperatively function for expression of genes involved in redox homeostasis, including GSH synthesis (Dickhout et al., 2012, Done and Traustadottir, 2016, Harding et al., 2003, Kasai et al., 2020, Lewerenz and Maher, 2009, Torrence et al., 2021, Ye et al., 2014). Therefore, changes of GSH/GSSG and ROS levels induced by ATF4 OE in *A/A* cells under ER stress conditions may be due to the transcriptional activity not only of ATF4 but also of Nrf2. Thus, our data imply that the increased sensitivity of PERK-knockout (Harding et al., 2003), eIF2α phosphorylation-deficient *A/A* (Back et al., 2009, Choi et al., 2017, Lewerenz and Maher, 2009), and ATF4-knockout (Dickhout et al., 2012, Harding et al., 2003) cells to oxidative stress is caused by deficiency of both ATF4 and Nrf2 expression.

Under ER stress conditions, eIF2α phosphorylation deficiency played a crucial role in the expression of genes involved in the serine-driven 1-carbon metabolism pathway, particularly those in the mitochondrial 1-carbon metabolism pathway (*Shmt2*, *Mthfd2*, *Mthfd1l*, and *Aldh1l2*) as shown in Fig. 3A-C. Moreover, eIF2α phosphorylation was necessary for the expression of the *Idh2*, *Me2*, and *Me3* genes, which encode the enzymes that produce NADPH during the oxidative decarboxylation of TCA cycle metabolites (Fig. 3A and B). As a result of the decreased expression of these genes, total NADP(H) levels in *A/A* MEFs under ER stress conditions were significantly lower than in *S/S* MEFs during ER stress (Fig. 3D). Mitochondria contain significantly higher levels of NADPH compared with the cytosol, although intracellular content of NADP(H) varies markedly among tissues and cell types (Tao et al., 2017). NADPH predominates over NADP^+^, with a ratio ranging from 15 to 333 (Hedeskov et al., 1987, Tao et al., 2017). Furthermore, it has been proposed that cytosolic and mitochondrial NADPH fluxes are regulated independently (Niu et al., 2023). Therefore, it is possible that impairment of NADPH homeostasis in *A/A* cells during ER stress was caused by dysregulated expression of mitochondrial NADPH-producing genes mentioned above. Furthermore, several reports suggest that interference with mitochondrial NADPH-producing genes, such as *Shmt2*, *Mthfd2*, and *Aldh1l2*, reduces mitochondrial NADPH production. This reduction increases levels of ROS and lipid peroxides, thereby enhancing susceptibility to ferroptosis or increasing the occurrence of ferroptosis (Du et al., 2024, Hennequart et al., 2023, Krupenko et al., 2020, Mo et al., 2024a, Mo et al., 2024b). Consequently, the reduction of both GSH and NADPH in the mitochondria of *A/A* cells under ER stress conditions may fail to suppress the accumulation of mitochondrial ROS and lipid peroxides, potentially leading to ferroptosis and apoptosis (see Graphical abstract).

Unexpectedly, in *A/A* cells, which can resolve NADPH homeostasis disruption through wild-type eIF2α OE (Figure 4D-F), this issue was not addressed by ATF4 OE under ER stress conditions (Figure 4A-C). The previously reported increase in the expression of genes related to the PPP and the serine-driven 1-carbon metabolism pathway associated with ATF4 (Wang et al., 2022, Xia et al., 2019, Zhao et al., 2020) was not observed. Furthermore, ATF4 OE further suppressed the expression of proteins such as SHMT2, MTHFD2, MTHFD1L, and ALDH1L2 in *A/A* cells during ER stress (Figure 4B). These unexpected phenomena can be explained in several ways. First, the effects of ATF4 on the expression of genes related to the PPP and the serine-driven 1-carbon metabolism pathway have been observed in tissues or cancer cells rather than in MEFs (Wang et al., 2022, Xia et al., 2019). Second, previous reports have documented gene expression changes induced by ATF4 under conditions involving mild stress, such as MYCN amplification or transverse aortic constriction (Wang et al., 2022, Xia et al., 2019), whereas the experimental conditions used in this study involve a robust ER stress environment. Third, ATF4 exerts its diverse regulatory roles through post-translational modifications and interactions with multiple heterodimerization partners (Neill and Masson, 2023). Additionally, its shared binding specificity with these dimerization partners on the promoters of target genes allows ATF4 to either cooperate with or compete against various TFs (Neill and Masson, 2023). However, these events may not occur in *A/A* cells lacking eIF2α phosphorylation under ER stress conditions. Consequently, the positive effects of ATF4 OE may not be evident in *A/A* cells under these conditions. Further investigation is required to address these issues.

The *Fsp*1 mRNA and its protein levels were significantly higher in *A/A* cells than in *S/S* cells, regardless of ER stress (Figs. 5D, 5E, S2F, and S2G). Intriguingly, ATF4 OE also slightly increased its mRNA and protein levels in *A/A* cells, regardless of ER stress (Figure 7A and B). Furthermore, the cell viability assays using iFSP1 revealed that increasing iFSP1 concentrations further reduced the viability of *A/A* cells under vehicle-treated conditions, as well as RSL3-treated (a ferroptosis activator at 0.5 and 2.5 µM, Fig. S9A) and Tm-treated (an ER stress inducer at 0.1 and 0.5 µg/ml, Fig. S9B) conditions. This implies that the increased level of FSP1 protects *A/A* cells from a certain degree of ferroptosis under ER stress conditions (up to 0.5 µg/ml Tm). However, cell death was not exacerbated upon FSP1 inhibition under the highest treatment conditions of RSL3 (5 µM, Fig. S9A) and Tm (1 µg/ml, Fig. S9B). This suggests that the elevated level of FSP1 is inadequate to counter the intense ferroptosis induced by 1 µg/ml Tm treatment in GSH-depleted *A/A* cells (Fig. 1E-I), alongside the dysregulated expression of ferroptosis-regulating genes (Figs. 5D, 5E, S2F, and S2G). In addition, FSP1 is a ferroptosis-related oxidoreductase that requires NADH/NADPH and CoQ10 (Jiang et al., 2021). Therefore, with increasing Tm concentrations, cells need to preserve or increase the levels of NADH/NADPH and CoQ10. Therefore, it is necessary to study whether *A/A* cells have sufficient amounts of NADH/NADPH and CoQ10 required for upregulated FSP1 protein under 1 µg/ml Tm-treated conditions. However, our results are insufficient to conclude whether FSP1 acts as an antiferroptotic protein under the Tm (1 µg/ml)-treated conditions used in all other experiments in this report. Thus, it is both interesting and scientifically important to understand why and how the levels of *Fsp1* mRNA and its protein in *A/A* cells are increased under normal conditions and whether the upregulation of FSP1 in *A/A* cells helps to mitigate ferroptosis under our experimental conditions. However, these studies are beyond the scope of this paper because our report focused on ATF4-mediated transcriptional reprogramming (not just FSP1 expression). Moreover, GSH replenishment effectively inhibited ferroptosis in *A/A* cells under ER stress conditions (Figure 6). Therefore, further studies are not necessary to draw the conclusions of this report.

Based on previous reports (Back et al., 2009, Cao and Kaufman, 2014, Choi et al., 2017, Han et al., 2013) and the data from Figs. S2E and S7B showing apoptotic cell death (such as TUNEL positivity and caspase-3 activation) in eIF2α phosphorylation-deficient cells and tissues under ER stress conditions, eIF2α phosphorylation is proposed to be important to alleviate ER stress-mediated apoptotic cell death. However, Annexin V and 7-AAD staining assays indicated that eIF2α phosphorylation is also required to suppress other forms of cell death involving the loss of plasma membrane integrity under ER stress conditions (Fig. S7C and D). Furthermore, the eIF2α phosphorylation-ATF4 axis helps to mitigate a newly discovered type of nonapoptotic cell death, ferroptosis (Cao and Dixon, 2016, Jiang et al., 2021, Stockwell, 2022), in response to ER stress (Figures 5 and 7). ATF4 in the axis (Young and Wek, 2016) promotes the transcriptional expression of GSH synthesis-related genes (*Cth*, *Gclc*, *Gsr*, *Slc3a2*, and *Slc7a11*) (Figures 1C, 2D, 2A, 2B, S2C, and S2D) and ferroptosis-suppressing genes (*Dhodh* and *Gpx4*) but suppresses the transcriptional expression of ferroptosis-activating genes (*Acsl4*, *Alox12*, and *Ptgs2*) (Figures 5D, 5E, 7A, 7B, S2F, and S2G) during ER stress. Consistently, eIF2α phosphorylation deficiency exacerbated GSH depletion (Fig. 1E-I, S1C, and S1D) and the accumulation of specific ferroptosis indicators (MDA and cellular and mitochondrial lipid peroxides) (Fig. 5F-H and S8D-F) during ER stress. Therefore, GSH supplementation and ATF4 OE effectively suppressed the accumulation of MDA (Fig. 6, Fig. 7) and cellular (Fig. 6, Fig. 7) and mitochondrial (Fig. 6, Fig. 7) lipid peroxides, thereby inhibiting ferroptotic cell death in Tm-treated *A/A* MEFs (Fig. 6, Fig. 7). However, GSH supplementation did not prevent apoptosis under ER stress conditions (Fig. S10E), even though it prevented the decrease in GPX4 protein levels under those conditions because the thiol group in GSH not only supports GPX4 activity but also enhances selenium-mediated GPX4 protein levels (Carlisle et al., 2020, Lee et al., 2021, Lee et al., 2024). On the other hand, ATF4 OE reduced apoptosis as well as ferroptosis under ER stress conditions, although the protective effect was not as strong as that of eIF2α (WT) OE (Figure 7F-J). Thus, the eIF2α phosphorylation-ATF4 axis was responsible for mitigating ferroptosis as well as apoptosis in *A/A* cells during ER stress.

There is some controversy regarding the role of DHODH in ferroptosis (Mao et al., 2023, Mao et al., 2021, Mishima et al., 2023). It is, therefore, possible that DHODH plays a limited role in the regulation of ferroptosis. Consequently, we do not claim that a decrease in DHODH levels can simply induce ferroptosis under ER stress conditions. Based on our data, we proposed that ferroptosis in *A/A* cells under ER stress is induced not only by changes in gene expression, such as the upregulation of ferroptosis-inhibiting genes and the downregulation of ferroptosis-activating genes but also by the decrease in levels of GSH and NADPH, both of which play important roles for inhibiting ferroptosis. Nevertheless, the expression of FSP1, a critical mediator of ferroptosis defense, was significantly increased in *A/A* cells, regardless of ER stress (Figs. 5D, 5E, S2F, and S2G). Additionally, our data regarding DHODH expression do not support the involvement of *A/A* cells in sensitization to ER stress inducers and ROS. Instead, we believe that decreased DHODH levels, combined with the reduced expression of other genes, particularly GPX4, and the reduction in mitochondrial GSH and NADPH levels, facilitate the induction of mitochondrial lipid peroxidation and subsequently ferroptosis during ER stress (see Graphical abstract), as Mao et al suggested that ferroptosis is induced by mitochondrial lipid peroxidation when expression of both DHODH and GPX4 is dysregulated (Figs. 5D, 5E, S2F, and S2G) (Mao et al., 2023). Therefore, we propose that deficiency in the eIF2α phosphorylation-ATF4 pathway leads to a decrease in DHODH levels (Figures 5D, 5E, 7A, 7B, S2F, and S2G), which may be one of the components necessary for inducing mitochondria-mediated ferroptosis under ER stress conditions. However, further studies are needed to determine how critical the decrease in DHODH expression is for the induction of ferroptosis in *A/A* cells triggered by ER stress. For instance, can the OE of DHODH in *A/A* cells inhibit mitochondrial lipid peroxidation and, subsequently, the induction of ferroptosis during ER stress?

In conclusion, we found that eIF2α phosphorylation suppresses the impairment of redox homeostasis (especially GSH and NADPH homeostasis) upon ER stress and protects cells from cell death, including ferroptosis, via transcriptional reprogramming induced by its downstream TFs. Therefore, targeting eIF2α phosphorylation may be a potential approach to treat rapidly growing tumors, which rely on UPR activation, mitochondrial functions, and redox homeostasis to adapt to stressful microenvironments (Chen and Cubillos-Ruiz, 2021, Wang and Kaufman, 2014).

## FUNDING AND SUPPORT

This work was funded by the Basic Science Research Program (2022R1A2C1010449 and RS-2024-00408826 to S.H.B.) of the National Research Foundation of Korea, which is funded by the Korean government.

## AUTHOR CONTRIBUTIONS

H.T.L., Y.K., M.K., S.H.H., and H.K. performed the experiments; H.T.L., Y.K., D.S., and S.H.B. analyzed the data; S.W.C., Y.J., H.T.C., and D.S. contributed reagents/materials/analysis tools. H.T.L., D.S., and S.H.B. wrote the paper; and H.T.L. and S.H.B. conceived and designed the experiments and critically reviewed the paper. All authors have read and approved the paper.

## CRediT Authorship Contribution Statement

**Le Hien Thi:** Writing – review and editing, Writing – original draft, Visualization, Validation, Methodology, Investigation, Data curation, Conceptualization. **Kim Mi-Jeong:** Visualization, Validation, Resources, Data curation. **Kim Yonghwan:** Writing – original draft, Visualization, Methodology, Data curation. **Kim Hyeeun:** Visualization, Validation, Resources, Formal analysis. **Hyun Seung Hwa:** Validation, Formal analysis, Data curation. **Chung Su Wol:** Resources, Methodology. **Chung Hun Taeg:** Resources, Methodology. **Joe Yeonsoo:** Resources, Methodology. **Back Sung Hoon:** Writing – review and editing, Writing – original draft, Supervision, Resources, Methodology, Investigation, Funding acquisition, Data curation, Conceptualization. **Shin Dong-Myung:** Writing – original draft, Resources, Methodology.

## DECLARATION OF COMPETING INTERESTS

No potential conflict of interest was reported by the authors.
