# Supplementary material for: Phosphorylation of eIF2α suppresses the impairment of GSH/NADPH homeostasis and mitigates the activation of cell death pathways, including ferroptosis, during ER stress
Source: Mol Cells. 2025 Mar 13;48(5):100210. doi: 10.1016/j.mocell.2025.100210 (PMC11999272; doi:10.1016/j.mocell.2025.100210)
Supplement: Supplementary file 1 — Supplementary material [file mmc1.pdf]

Supplementary Materials

Phosphorylation of eIF2α suppresses the impairment of GSH/NADPH homeostasis and mitigates the activation of cell death pathways, including ferroptosis, during ER stress

Supple Fig-01

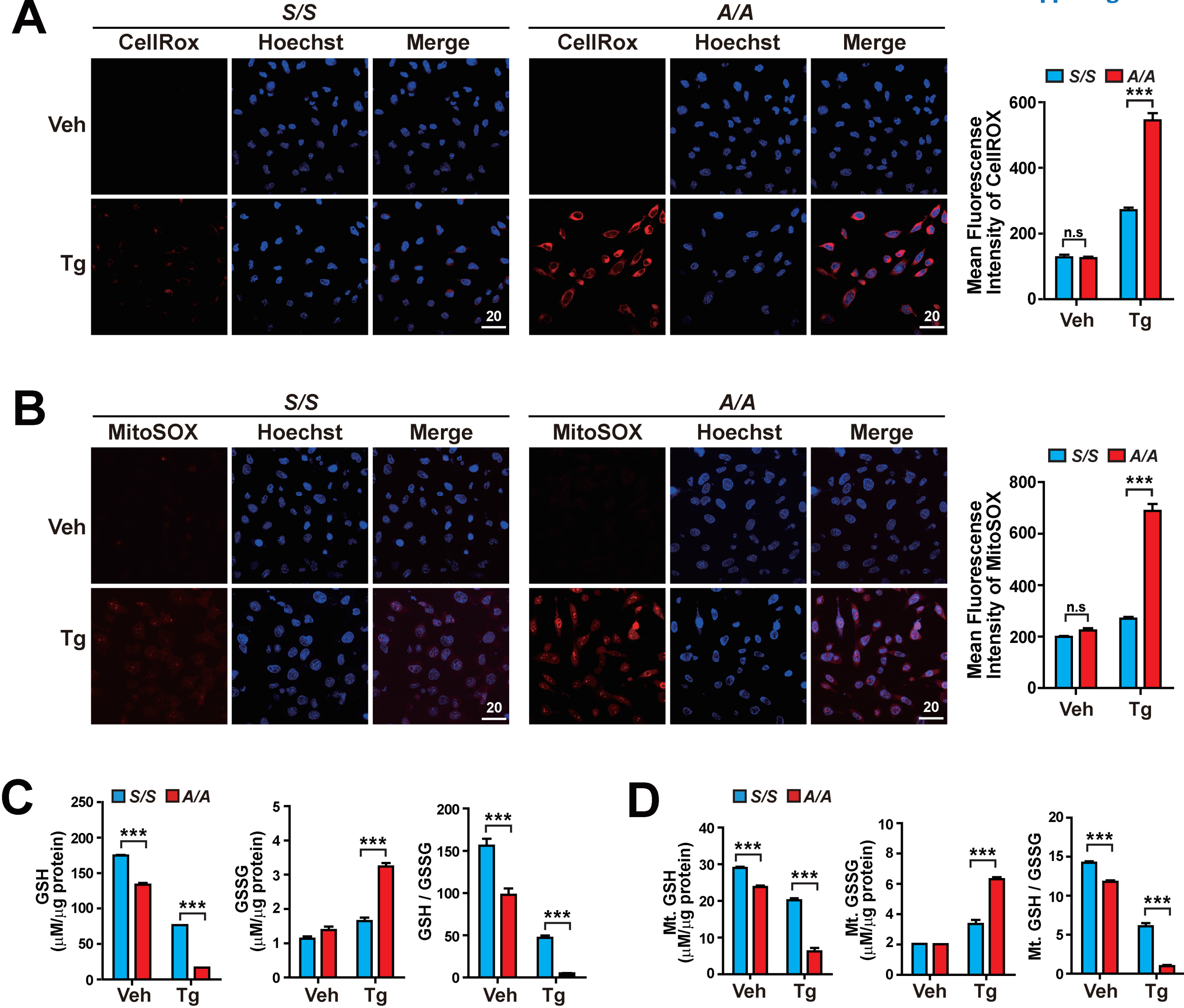

Figure S1. eIF2α phosphorylation deficiency leads to accumulation of GSH depletion, as well as cellular and mitochondrial ROS in Tg-treated MEFs. (A and B) Representative CellROX and MitoSOX staining images of *S/S* and *A/A* MEFs. Cells were treated with Veh (DMSO) or Tg (500 nM) for 24 h and stained with CellROX Deep Red (red) and Hoechst 33258 (blue) or MitoSOX Red (red) and Hoechst 33258 (blue) for the last 30 min. Scale bar: 20 μm. The graphs depict quantification of the MFI of CellROX or MitoSOX. Data are presented as mean ± SEM (n = 3, 15 random fields per condition). ***p < 0.001, *S/S* vs. *A/A*. **(C)** Quantification of cellular GSH and GSSG levels in *S/S* and *A/A* MEFs treated with Veh or Tg for 24 h. Data are presented as mean ± SEM (n = 3). ***p < 0.001, *S/S* vs. *A/A*. **(D)** Quantification of mitochondrial (Mt) GSH and GSSG levels in mitochondria-enriched fractions of *S/S* and *A/A* MEFs treated with Veh or Tg for 24 h. Data are presented as mean ± SEM (n = 3). ***p < 0.001, *S/S* vs. *A/A*.

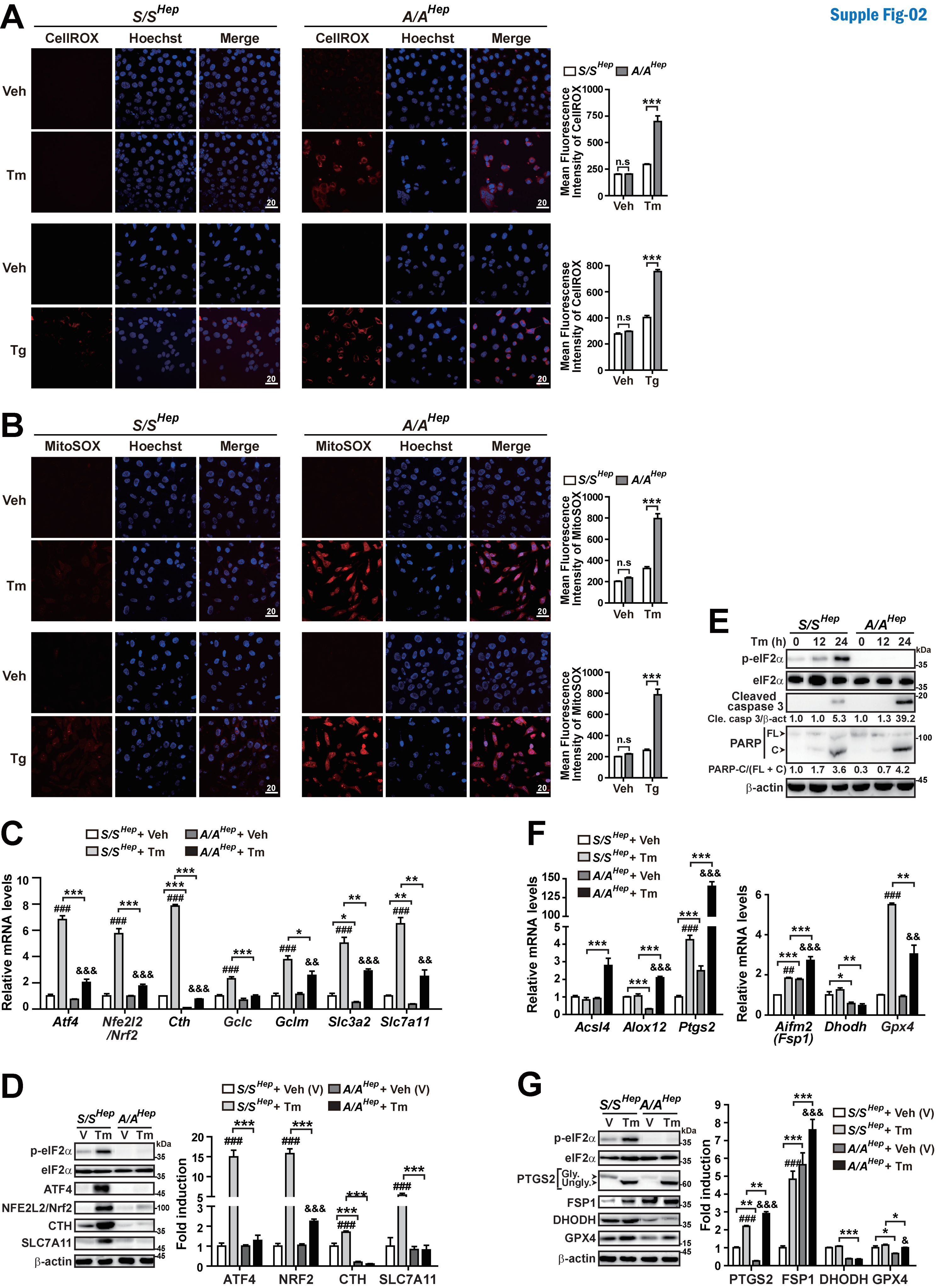
A
S/S^Hep
A/A^Hep
CellROX
Hoechst
Merge
Veh
Tm
Tg
20
Mean Fluorescense Intensity of CellROX
B
MitoSOX
Mean Fluorescense Intensity of MitoSOX
n.s
***
C
S/S^Hep + Veh
S/S^Hep + Tm
A/A^Hep + Veh
A/A^Hep + Tm
Relative mRNA levels
Atf4
Nfe2l2 /Nrf2
Cth
Gclc
Gclm
Slc3a2
Slc7a11
D
p-eIF2α
eIF2α
ATF4
NFE2L2/Nrf2
CTH
SLC7A11
β-actin
Fold induction
ATF4
NRF2
CTH
SLC7A11
E
Tm (h) 0 12 24 0 12 24
Cleaved caspase 3
Cle. casp 3/β-act 1.0 1.0 5.3 1.0 1.3 39.2
PARP
FL
C
PARP-C/(FL + C) 1.0 1.7 3.6 0.3 0.7 4.2
F
Acsl4
Alox12
Ptgs2
Aifm2 (Fsp1)
Dhodh
Gpx4
G
PTGS2
Gly.
Ungly.
FSP1
DHODH
GPX4
kDa

Figure S2. eIF2α phosphorylation is required for optimal expression of GSH- and ferroptosis-related genes and thereby prevents ROS accumulation and ferroptosis during ER stress. (A and B) Representative CellROX and MitoSOX staining images of S/S^{Hep} and A/A^{Hep} cells. Cells were treated with Veh, Tm, or Tg for 24 h and stained with CellROX Deep Red (red) and Hoechst 33258 (blue) or MitoSOX Red (red) and Hoechst 33258 (blue) for the last 30 min. Scale bar: 20 µm. The graphs depict quantification of the MFI of CellROX or MitoSOX. Data are presented as mean ± SEM (n = 3, 15 random fields per condition). ***p < 0.001, S/S^{Hep} vs. A/A^{Hep}. **(C)** Quantitative RT-PCR analysis of mRNA levels of GSH-related genes in S/S^{Hep} and A/A^{Hep} cells treated with Veh or Tm for 24 h. **(D)** WB analysis of GSH-related proteins (ATF4, CTH, and SLC7A11) in lysates of S/S^{Hep} and A/A^{Hep} cells treated with Veh or Tm for 24 h. The graph depicts the protein level normalized by the β-act level. **(E)** WB analysis of cleaved caspase 3 and PARP in lysates of S/S^{Hep} and A/A^{Hep} cells treated with Tm for the indicated durations. Protein levels normalized by β-act or the indicated protein levels are shown below the panels. FL, full-length protein; C, C-terminal fragment; N, N-terminal fragment. **(F)** Quantitative RT-PCR analysis of mRNA levels of ferroptosis-activating (upper graph) and ferroptosis-inhibiting (lower graph) genes in S/S^{Hep} and A/A^{Hep} cells treated with Veh or Tm for 24 h. **(G)** WB analysis of ferroptosis-related proteins (PTGS2, FSP1, DHODH, and GPX4) in lysates of S/S^{Hep} and A/A^{Hep} cells treated with Veh or Tm for 24 h. PTGS2 was deglycosylated by Tm treatment. Gly, glycosylated PTGS2; Ungly, unglycosylated PTGS2. The graph depicts the protein level normalized by the β-act level. Data are presented as mean ± SEM (n = 3). *p < 0.05, **p < 0.01, and ***p < 0.001, S/S^{Hep} vs. A/A^{Hep}; $^{\#}$p < 0.05, $^{\#\#}$p

< 0.01, and $^{\#\#\#}p < 0.001$, Veh vs. Tm in S/S^{Hep}; $^{\&}p < 0.05$, $^{\&\&}p < 0.01$, and $^{\&\&\&}p < 0.001$, Veh vs. Tm in A/A^{Hep}.

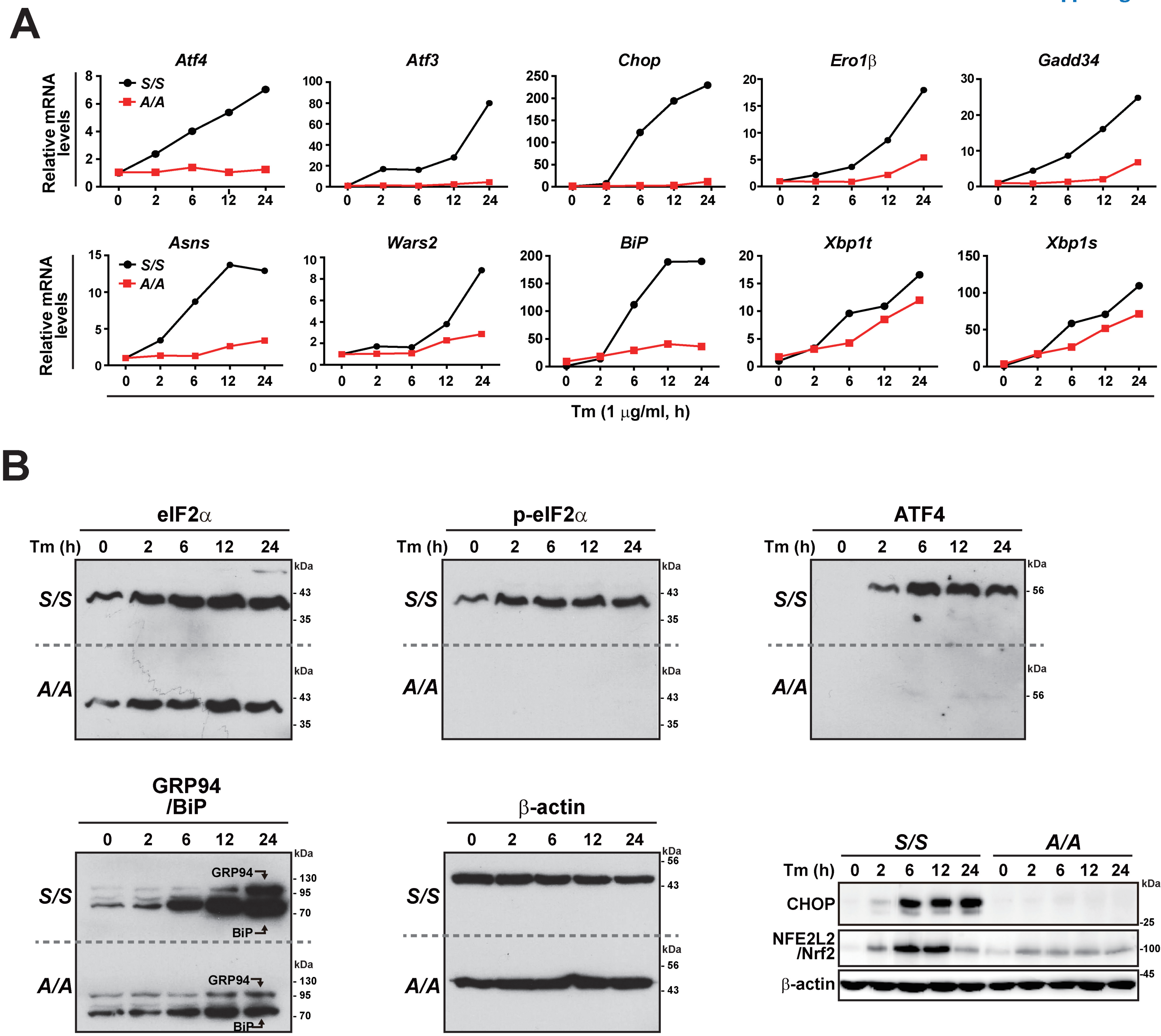
A
Atf4
Atf3
Chop
Ero1β
Gadd34
Asns
Wars2
BiP
Xbp1t
Xbp1s
Relative mRNA levels
S/S
A/A
Tm (1 μg/ml, h)
B
eIF2α
p-eIF2α
ATF4
GRP94 /BiP
β-actin
Tm (h)
0
2
6
12
24
kDa
S/S
A/A
GRP94
BiP
CHOP
NFE2L2 /Nrf2
β-actin

Figure S3. Expression of multiple UPR genes is dysregulated in *A/A* MEFs during ER stress. (A) Quantitative RT-PCR analysis of mRNA levels of several UPR genes in *S/S* and *A/A* MEFs treated with Tm for the indicated durations. **(B)** WB analysis of p-eIF2α, ATF4, and its downstream proteins in lysates of *S/S* and *A/A* MEFs treated with Tm for the indicated durations.

Supple Fig-04

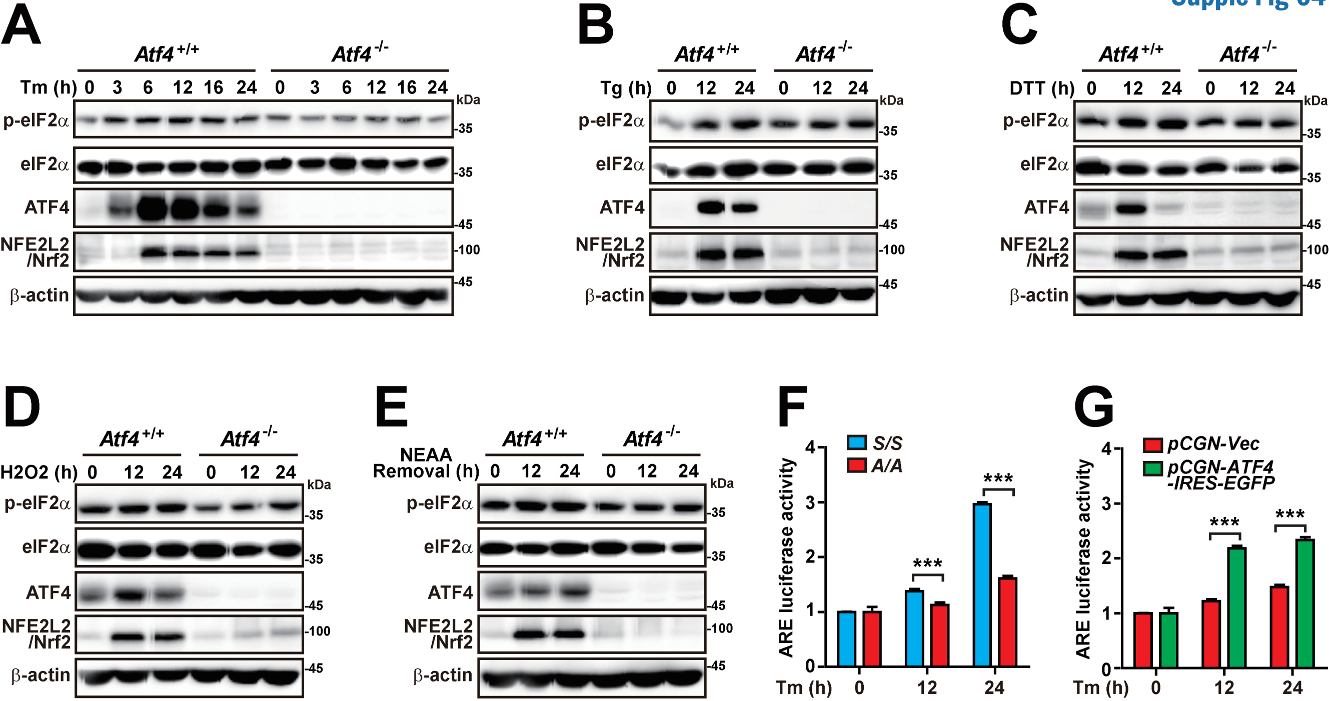

Figure S4. ATF4 is required for NFE2L2/Nrf2 expression under several cellular stress conditions. (A–D) WB analysis of p-eIF2α, eIF2α, ATF4, and NFE2L2/Nrf2 in lysates of *ATF4*$^{+/+}$ and *ATF4*$^{-/-}$ MEFs treated with ER stress inducers [(A) Tm (1 μg/mL), (B) Tg (500 nM), and (C) DTT (2 mM)] or (D) the oxidative stress inducer H_2O_2 (500 μM) for the indicated durations. **(E)** WB analysis of p-eIF2α, eIF2α, ATF4, and Nrf2 in lysates of *ATF4*$^{+/+}$ and *ATF4*$^{-/-}$ MEFs cultivated without addition of NEAAs for the indicated durations. **(F)** Luciferase activity assay of the Nrf2 response element (**Antioxidant Response Element,** ARE) luciferase reporter in *S/S* and *A/A* MEFs. MEFs were co-transfected with *pGL4.37-ARE-firefly luciferase* and *pRL-CMV* for 24 h. Cells were then treated with Tm for the indicated durations and luciferase activities were measured. Data are presented as mean ± SEM (n = 3). ***p < 0.001, *S/S* vs. *A/A*. **(G)** Luciferase activity assay of the ARE luciferase reporter in ATF4-overexpressing *A/A* MEFs. MEFs were co-transfected with *pGL4.37-ARE-firefly luciferase*, *pRL-CMV*, and either *pCGN-Vec* or *pCGN-ATF4-IRES-EGFP* for 24 h. Cells were then treated with Tm for the indicated durations and luciferase activities were measured. Data are presented as mean ± SEM (n = 3). ***p < 0.001, *pCGN-Vec* vs. *pCGN-ATF4-IRES-EGFP*.

Supple Fig-05

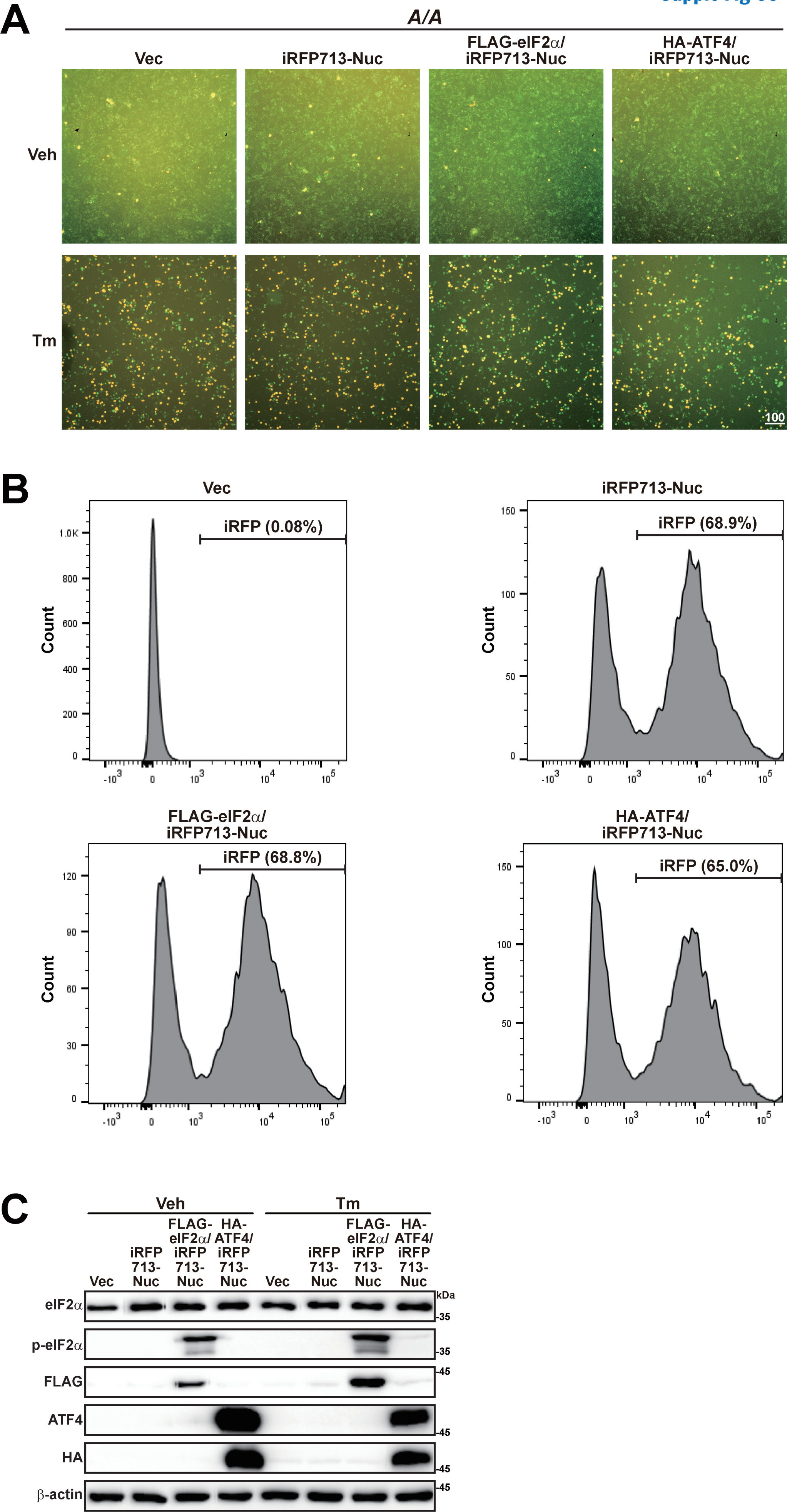

Figure S5. eIF2α or ATF4 OE suppresses the decrease of mitochondrial GSH levels in *A/A* cells during ER stress. (A) Representative merged images of F_{510} (green) and F_{580} (yellow) fluorescence in MitoFreSHtracer-loaded *A/A* MEFs. *A/A* MEFs were transfected with a plasmid expressing Vec, iRFP713-Nuc, FLAG-eIF2α/iRFP713-Nuc, or HA-ATF4/iRFP713-Nuc for 48 h. The cells were then treated with Veh or Tm for 24 h and stained with MitoFreSHtracer for the last 1 h. Scale bar: 100 µm. **(B)** Flow cytometric analysis of the transfection efficiency of plasmids expressing Vec, iRFP713-Nuc, FLAG-eIF2α/iRFP713-Nuc, or HA-ATF4/iRFP713-Nuc in *A/A* MEFs. The graph depicts the percentage of cells positive for far-red fluorescence (emission wavelength, 713 nm), i.e., iRFP713-Nuc-positive cells, determined using a flow cytometer. Data are presented as mean ± SEM (n = 3). **(C)** WB analysis of FLAG-eIF2α and HA-ATF4 in lysates of Vec-, iRFP713-Nuc-, FLAG-eIF2α/iRFP713-Nuc-, or HA-ATF4/iRFP713-Nuc-overexpressing *A/A* MEFs treated with Veh or Tm for 24 h.

Supple Fig-06

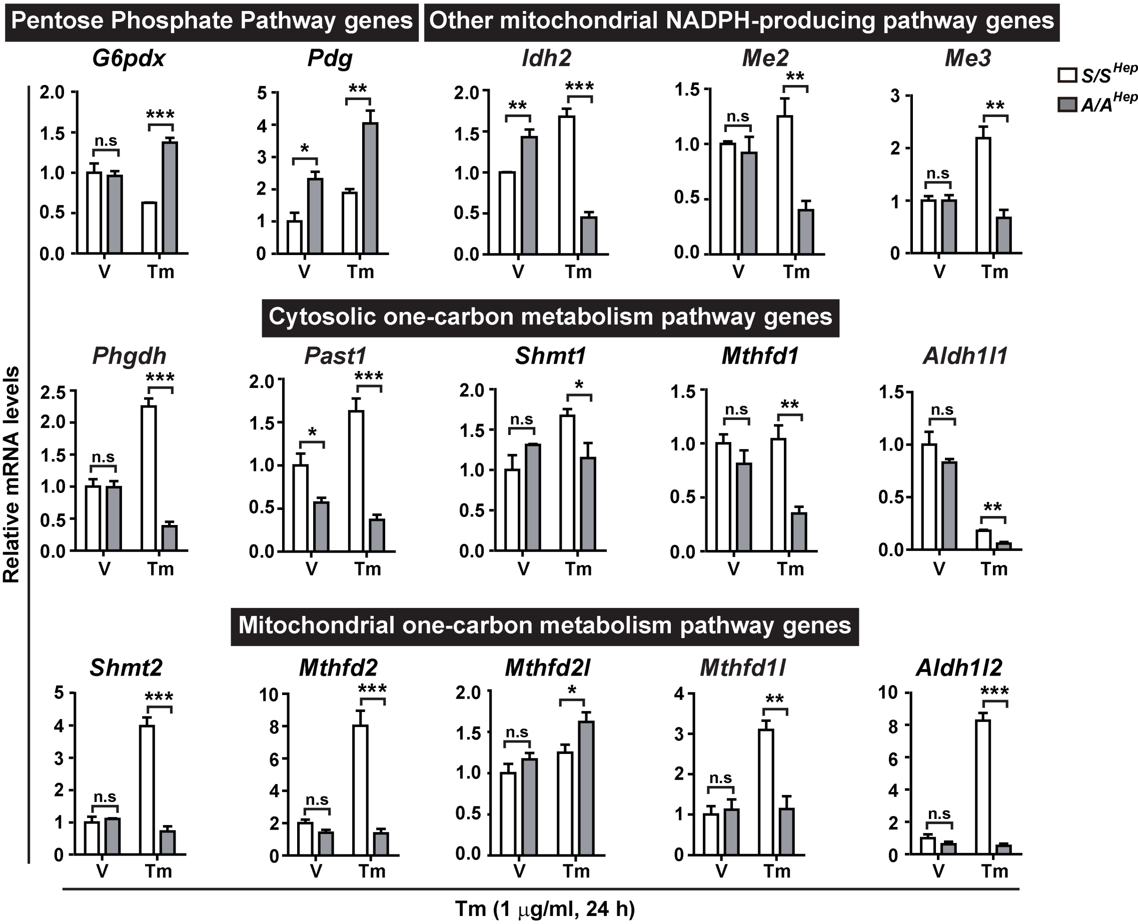

Figure S6. eIF2α phosphorylation deficiency dysregulates the expression of NADPH-producing genes in *A/A*^{Hep} during ER stress. Quantitative RT-PCR analysis of the mRNA levels of pentose phosphate pathway (PPP) genes, cytosolic one-carbon metabolism pathway gene, mitochondrial one-carbon metabolism pathway genes, and other mitochondrial NADPH-producing pathway genes in *S/S*^{Hep} *and A/A*^{Hep} *cells* treated with vehicle (V) or Tm (1 μg/mL) for 24 h. Data are presented as mean ± SEM (n = 3). **P* < .05, ***P* < .01, and ****P* < .001, *S/S*^{Hep} versus *A/A*^{Hep}.

Supple Fig-7

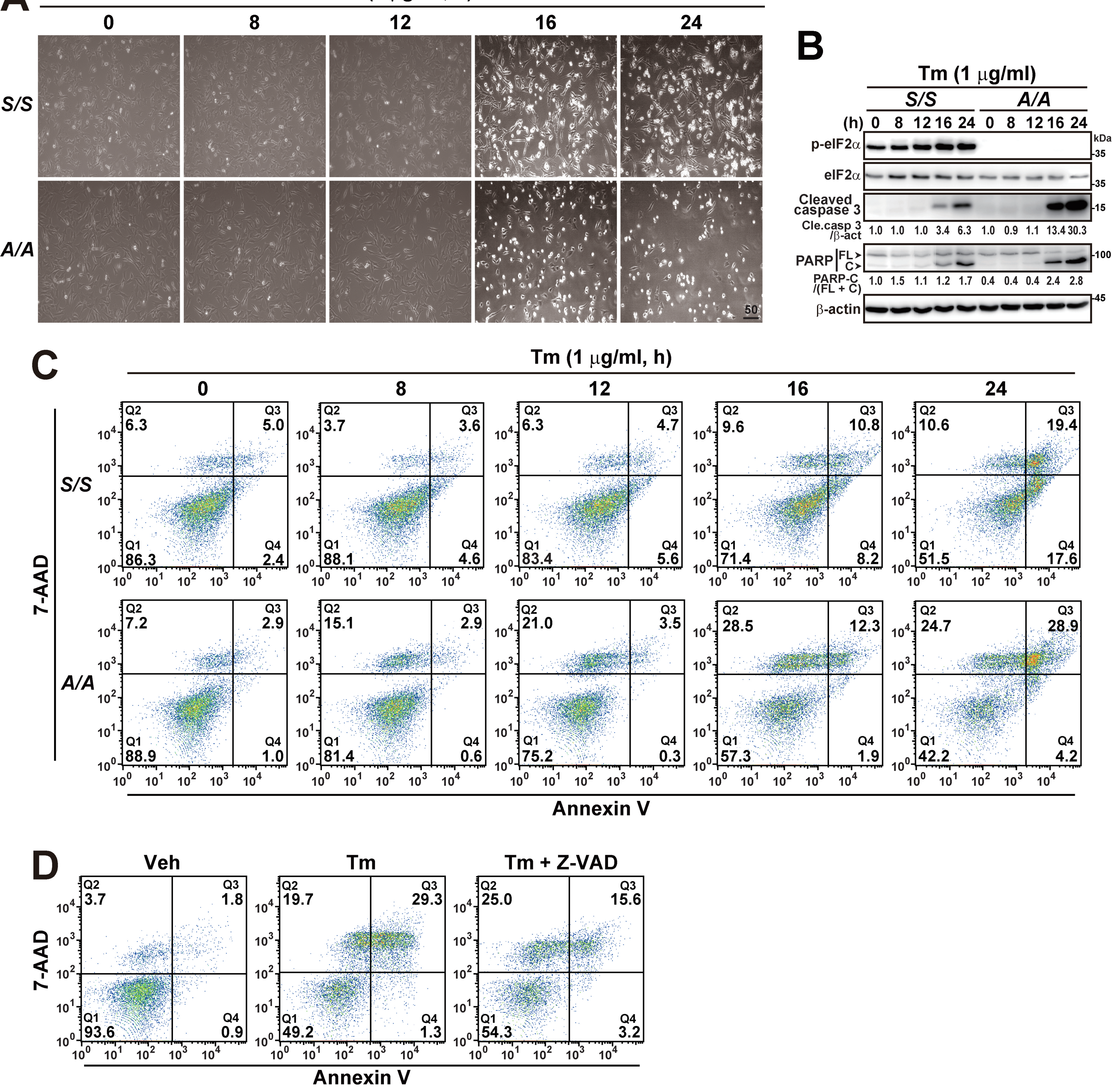

A
Tm (1 μg/ml, h)
0
8
12
16
24
S/S
A/A
50
B
Tm (1 μg/ml)
S/S
A/A
(h) 0 8 12 16 24 0 8 12 16 24
p-eIF2α
eIF2α
Cleaved caspase 3
Cle.casp 3 /β-act 1.0 1.0 1.0 3.4 6.3 1.0 0.9 1.1 13.4 30.3
PARP FL C
PARP-C /(FL + C) 1.0 1.5 1.1 1.2 1.7 0.4 0.4 0.4 2.4 2.8
β-actin
kDa
35
35
15
100
45
C
Tm (1 μg/ml, h)
0
8
12
16
24
S/S
A/A
7-AAD
Annexin V
Q1
Q2
Q3
Q4
S/S 0 h: Q2 6.3, Q3 5.0, Q1 86.3, Q4 2.4
S/S 8 h: Q2 3.7, Q3 3.6, Q1 88.1, Q4 4.6
S/S 12 h: Q2 6.3, Q3 4.7, Q1 83.4, Q4 5.6
S/S 16 h: Q2 9.6, Q3 10.8, Q1 71.4, Q4 8.2
S/S 24 h: Q2 10.6, Q3 19.4, Q1 51.5, Q4 17.6
A/A 0 h: Q2 7.2, Q3 2.9, Q1 88.9, Q4 1.0
A/A 8 h: Q2 15.1, Q3 2.9, Q1 81.4, Q4 0.6
A/A 12 h: Q2 21.0, Q3 3.5, Q1 75.2, Q4 0.3
A/A 16 h: Q2 28.5, Q3 12.3, Q1 57.3, Q4 1.9
A/A 24 h: Q2 24.7, Q3 28.9, Q1 42.2, Q4 4.2
D
Veh
Tm
Tm + Z-VAD
7-AAD
Annexin V
Veh: Q2 3.7, Q3 1.8, Q1 93.6, Q4 0.9
Tm: Q2 19.7, Q3 29.3, Q1 49.2, Q4 1.3
Tm + Z-VAD: Q2 25.0, Q3 15.6, Q1 54.3, Q4 3.2

Figure S7. eIF2α phosphorylation deficiency exacerbates not only apoptosis and but also other forms of cell death involving the loss of plasma membrane integrity under ER stress conditions. (A) Representative microscopic images of *S/S* and *A/A* MEFs treated with Tm for the indicated durations. Cells were observed under an inverted light microscope. Scale bar: 50 µm. **(B)** WB analysis of cleaved caspase 3 and PARP in lysates of *S/S* and *A/A* MEFs treated with Tm for the indicated durations. Protein levels normalized by β-act or the indicated protein levels are shown below the panels. **(C)** Representative flow cytometric analysis images of Annexin V- and 7-AAD-stained *S/S* and *A/A* MEFs treated with Tm for the indicated durations. The values shown represent the means (%) (n = 3). **(D)** Representative flow cytometric analysis images of Annexin V- and 7-AAD-stained *A/A* MEFs treated with Veh, Tm, or Tm plus Z-VAD (20 µM) for 24 h. The values shown represent the means (%) (n = 3).

Supple Fig-8

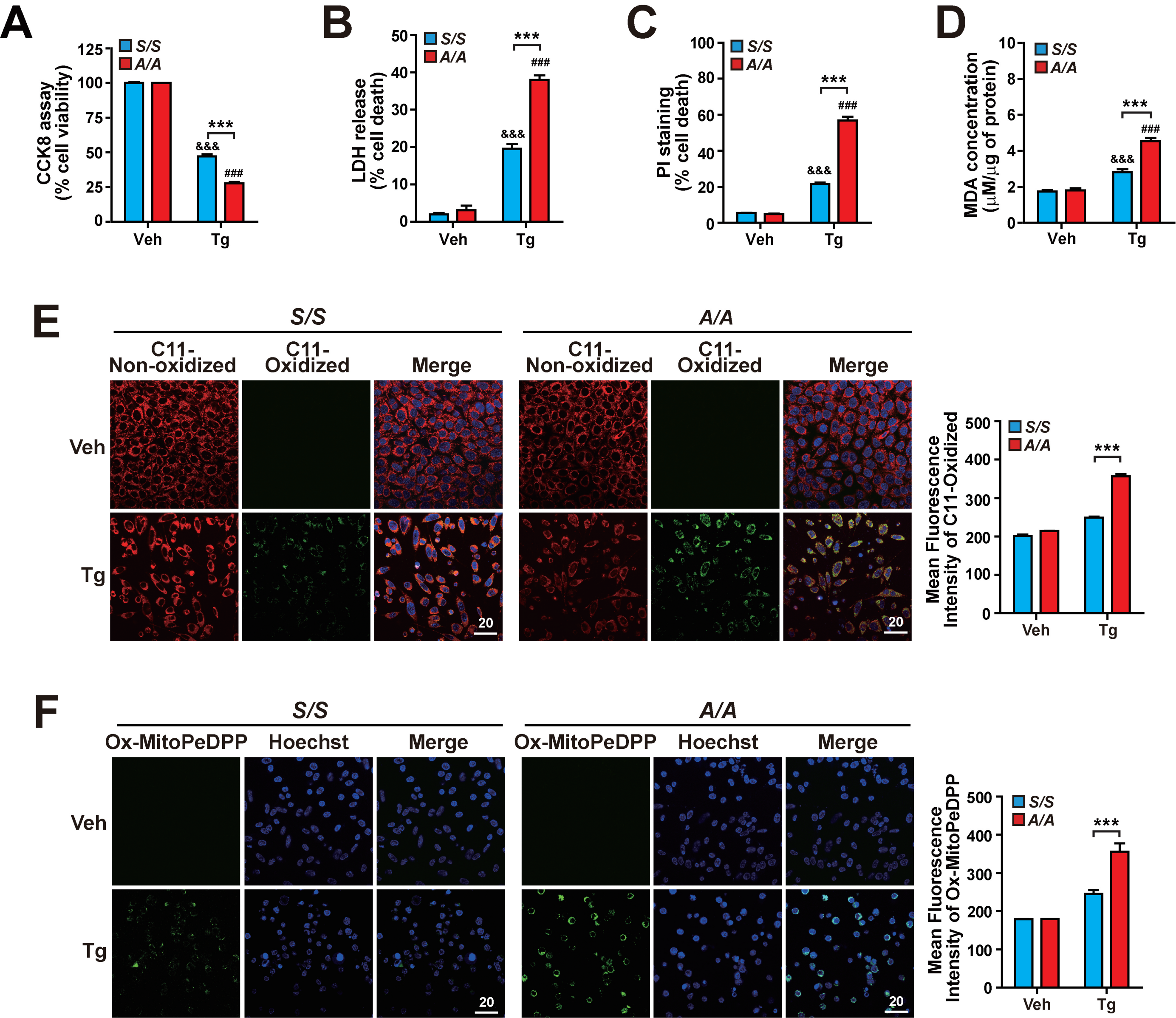
A
CCK8 assay (% cell viability)
S/S
A/A
Veh
Tg
***
&&&
###
B
LDH release (% cell death)
C
PI staining (% cell death)
D
MDA concentration (μM/μg of protein)
E
C11-Non-oxidized
C11-Oxidized
Merge
20
Mean Fluorescence Intensity of C11-Oxidized
F
Ox-MitoPeDPP
Hoechst
Mean Fluorescence Intensity of Ox-MitoPeDPP

Figure S8. eIF2α phosphorylation deficiency exacerbates cell death and the accumulation of lipid peroxides within both intracellular and mitochondrial compartments under Tg-treated conditions. (A–C) Cell viability measurements by the CCK-8 assay (A), cell death measurements by the LDH release assay (B), and PI staining (C, flow cytometric analysis) in *S/S* and *A/A* MEFs treated with Veh (24 h) or Tg for 24 h. Data are presented as mean ± SEM (n = 3). $^{***}p < 0.001$, *S/S* vs. *A/A*; $^{\&\&\&}p < 0.001$, Veh vs. Tg in *S/S*; $^{\#\#\#}p < 0.001$, Veh vs. Tg in *A/A*. **(D)** Measurement of MDA levels in *S/S* and *A/A* MEFs treated with Veh or Tg for 24 h. Data are presented as mean ± SEM (n = 3). $^{***}p < 0.001$, *S/S* vs. *A/A*; $^{\&\&\&}p < 0.001$, Veh vs. Tg in *S/S*; $^{\#\#\#}p < 0.001$, Veh vs. Tg in *A/A*. **(E and F)** Representative BODIPY 581/591 C11 staining images of *S/S* and *A/A* MEFs. Cells were treated with Veh or Tg for 24 h and stained with BODIPY 581/591 C11 (red and green) and Hoechst 33258 (blue) or MitoPeDPP (green) and Hoechst 33258 (blue) for the last 30 min. Scale bar: 20 µm. The graphs depict quantification of the MFI of C11-Oxidized or Ox-MitoPeDPP. Data are presented as mean ± SEM (n = 3, 15 random fields per condition). $^{***}p < 0.001$, *S/S* vs. *A/A*.

Supple Fig-9

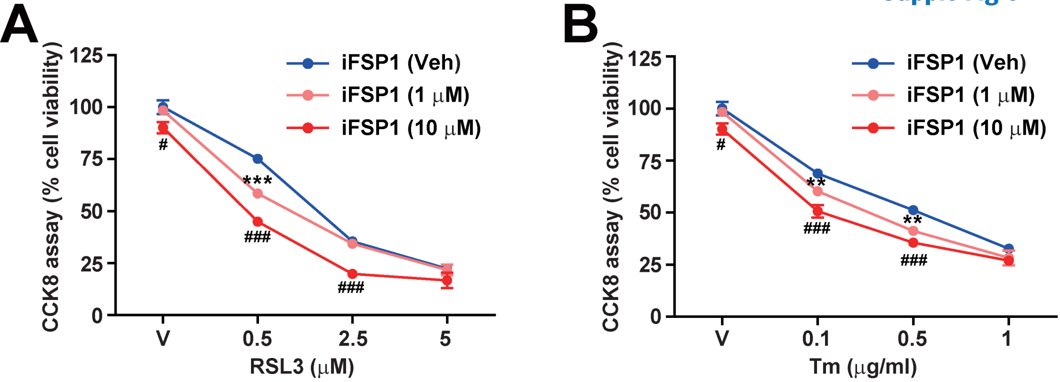
A
CCK8 assay (% cell viability)
125
100
75
50
25
0
iFSP1 (Veh)
iFSP1 (1 μM)
iFSP1 (10 μM)
#
***
###
###
V
0.5
2.5
5
RSL3 (μM)
B
CCK8 assay (% cell viability)
125
100
75
50
25
0
iFSP1 (Veh)
iFSP1 (1 μM)
iFSP1 (10 μM)
#
**
###
**
###
V
0.1
0.5
1
Tm (μg/ml)

Figure S9. The increased level of FSP1 protects *A/A* cells against a certain degree of ferroptosis and ER stress. (A and B) Cell viability measurements by the CCK-8 assay. *A/A* MEFs were pre-treated with three doses of iFSP1 for 2 h and then, co-incubated with the indicated doses of iFSP1 plus RSL3 or iFSP1 plus Tm for an additional 24 h. Data are presented as mean ± SEM (n = 3). $^{**}p < 0.01$ and $^{***}p < 0.001$, Veh vs. iFSP1 (1 μM); $^{\#}p < 0.05$ and $^{\#\#\#}p < 0.001$, Veh vs. iFSP1 (10 μM).

Supple Fig-10

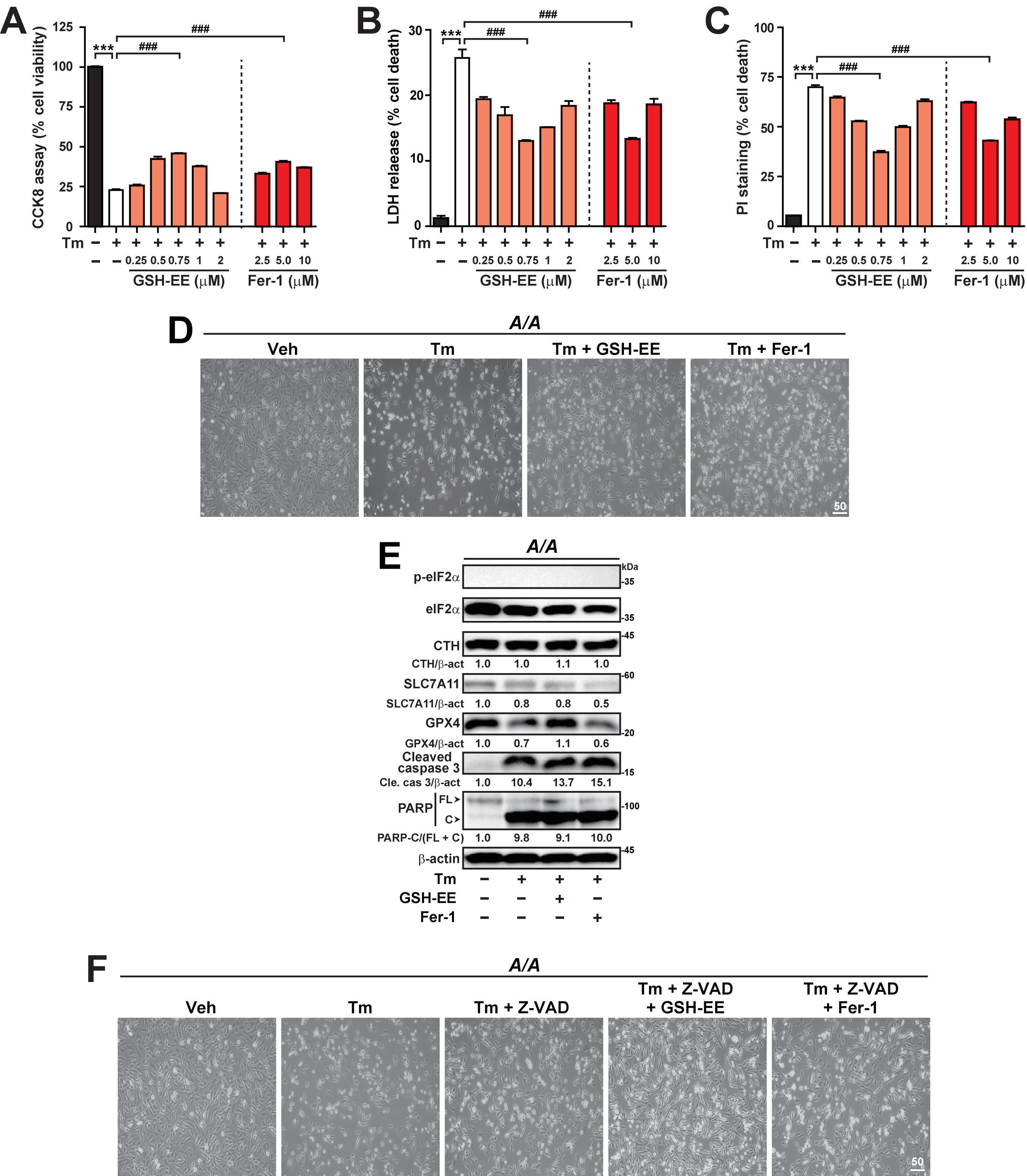

Figure S10. GSH supplementation suppresses ferroptosis but not apoptosis in Tm-treated *A/A* cells. (A–C) Cell viability measurements by the CCK-8 assay (A), cell death measurements by the LDH release assay (B), and PI staining (C) in *A/A* MEFs treated with Veh (DMSO, -), Tm, Tm plus GSH-EE, or Tm plus Fer-1 at the indicated concentrations for 24 h. Prior to treatment with Tm, Tm plus GSH-EE, or Tm plus Fer-1, cells were pre-treated with Veh (-), GSH-EE, or Fer-1 at the indicated concentrations for 1 h. Data are presented as mean ± SEM (n = 3). ***$p < 0.001$, Veh (-) vs. Tm; $^{###}p < 0.001$, Tm vs. either Tm plus GSH-EE or Tm plus Fer-1 at each concentration. **(D)** Representative microscopic images of *A/A* MEFs treated with Veh, Tm, Tm plus GSH-EE (0.75 μM), or Tm plus Fer-1 (5 μM) for 24 h. Prior to treatment with Tm, Tm plus GSH-EE, or Tm plus Fer-1, cells were pre-treated with Veh (-), GSH-EE, or Fer-1 for 1 h. Cells were observed under an inverted light microscope. Scale bar: 50 μm. **(E)** WB analysis of ferroptosis-suppressing proteins (CTH, SLC7A11, and GPX4) and apoptosis marker proteins (cleaved caspase 3 and PARP) in lysates of *A/A* MEFs treated Veh (DMSO, -), Tm, Tm plus GSH-EE, or Tm plus Fer-1 for 24 h. Prior to treatment with Tm, Tm plus GSH-EE, or Tm plus Fer-1, cells were pre-treated with Veh (-), GSH-EE, or Fer-1 at the indicated concentrations for 1 h. Protein levels normalized by β-act or the indicated protein levels are shown below the panels. **(F)** Representative microscopic images of *A/A* MEFs treated with Veh, Tm, Tm plus Z-VAD (20 μM), Tm plus Z-VAD (20 μM) plus GSH-EE (0.75 μM), or Tm plus Z-VAD (20 μM) plus Fer-1 (5 μM) for 24 h. Prior to treatment with Tm or Tm plus the indicated chemicals, cells were pre-treated with Veh (-), Z-VAD, Z-VAD plus GSH-EE, or Z-VAD plus Fer-1 for 1 h. Cells were observed under an inverted light microscope. Scale bar: 50 μm.
